# Mitochondrial dysfunction induced by HIF‐1α under hypoxia contributes to the development of gastric mucosal lesions

**DOI:** 10.1002/ctm2.1653

**Published:** 2024-04-15

**Authors:** Yuelin Xiao, Xianzhi Liu, Kaiduan Xie, Jiajie Luo, Yiwang Zhang, Xiaoli Huang, Jinni Luo, Siwei Tan

**Affiliations:** ^1^ Department of Gastroenterology The Third Affiliated Hospital of Sun Yat‐Sen University Guangzhou China; ^2^ Department of Pathology The Third Affiliated Hospital of Sun Yat‐Sen University Guangzhou China

**Keywords:** Drp1, gastric mucosal lesions, glycolysis, HIF‐1α, METTL3, mitochondrial dysfunction, mitochondrial fission, NLRP3

## Abstract

**Introduction:**

Hypoxia is an important characteristic of gastric mucosal diseases, and hypoxia‐inducible factor‐1α (HIF‐1α) contributes to microenvironment disturbance and metabolic spectrum abnormalities. However, the underlying mechanism of HIF‐1α and its association with mitochondrial dysfunction in gastric mucosal lesions under hypoxia have not been fully clarified.

**Objectives:**

To evaluate the effects of hypoxia‐induced HIF‐1α on the development of gastric mucosal lesions.

**Methods:**

Portal hypertensive gastropathy (PHG) and gastric cancer (GC) were selected as representative diseases of benign and malignant gastric lesions, respectively. Gastric tissues from patients diagnosed with the above diseases were collected. Portal hypertension (PHT)‐induced mouse models in *METTL3* mutant or *NLRP3*‐deficient littermates were established, and nude mouse gastric graft tumour models with relevant inhibitors were generated. The mechanisms underlying hypoxic condition, mitochondrial dysfunction and metabolic alterations in gastric mucosal lesions were further analysed.

**Results:**

HIF‐1α, which can mediate mitochondrial dysfunction via upregulation of METTL3/IGF2BP3‐dependent dynamin‐related protein 1 (Drp1) N6‐methyladenosine modification to increase mitochondrial reactive oxygen species (mtROS) production, was elevated under hypoxic conditions in human and mouse portal hypertensive gastric mucosa and GC tissues. While blocking HIF‐1α with PX‐478, inhibiting Drp1‐dependent mitochondrial fission via mitochondrial division inhibitor 1 (Mdivi‐1) treatment or *METTL3* mutation alleviated this process. Furthermore, HIF‐1α influenced energy metabolism by enhancing glycolysis via lactate dehydrogenase A. In addition, HIF‐1α‐induced Drp1‐dependent mitochondrial fission also enhanced glycolysis. Drp1‐dependent mitochondrial fission and enhanced glycolysis were associated with alterations in antioxidant enzyme activity and dysfunction of the mitochondrial electron transport chain, resulting in massive mtROS production, which was needed for activation of NLRP3 inflammasome to aggravate the development of the PHG and GC.

**Conclusions:**

Under hypoxic conditions, HIF‐1α enhances mitochondrial dysfunction via Drp1‐dependent mitochondrial fission and influences the metabolic profile by altering glycolysis to increase mtROS production, which can trigger NLRP3 inflammasome activation and mucosal microenvironment alterations to contribute to the development of benign and malignant gastric mucosal lesions.

## Graphical abstract

**Highlights**

- Hypoxia‐inducible factor‐1α (HIF‐1α) upregulation under hypoxia promotes mitochondrial fission/dysfunction via METTL3/IGF2BP3‐mediated dynamin‐related protein 1 (Drp1) N6‐methyladenosine methylation and enhances glycolysis via lactate dehydrogenase A in both portal hypertensive gastropathy and gastric cancer.
- Drp1‐dependent mitochondrial fission/dysfunction and enhanced glycolysis are associated with alterations in antioxidant enzyme activity and dysfunction of the mitochondrial electron transport chain, resulting in massive mitochondrial reactive oxygen species (mtROS) production.
- Massive mtROS production leads to NLRP3 inflammasome activation to promote the development of gastric mucosal lesions.
- Hypoxia repair or targeting of the HIF‐1α‐related pathway may be effective treatments for gastric mucosal lesions under hypoxic condition.

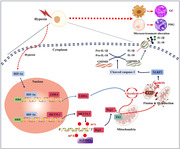

## 1 INTRODUCTION

Oxygen is essential for normal aerobic metabolism in mammals. Hypoxia is characterised by impaired oxygen delivery or uptake/utilisation and is related to disease progression, resistance to conventional therapies and poor prognosis.^1^ Hypoxia is crucial to gastric mucosal lesions and the development of gastric mucosal diseases, especially portal hypertensive gastropathy (PHG) and gastric cancer (GC), may be related to alterations in the mucosal microenvironment.^2^ PHG, a significant complication of portal hypertension (PHT),^3^ is thought to be a vascular illness with a convoluted aetiology induced by humoral factors, splanchnic blood flow, regional abnormalities in the control of vascular tone, etc.^4^, ^5^ Currently, the association between organic hypoxic conditions and alterations in the microenvironment of the gastric mucosa needs to be clarified. Hypoxia‐inducible factor‐1α (HIF‐1α), together with HIF‐1β, is a hypoxia‐inducible factor that regulates hypoxia‐inducible genes.^6^, ^7^ Functional studies revealed that damage to the gastric mucosa occurs via the modulation of HIF‐1α signalling, which is especially involved in GC.^8^ However, how increased HIF‐1α levels, in response to hypoxia in PHT and GC, lead to pathological changes in the gastric mucosa remains to be determined.

Hypoxia leads to oxygen delivery and consumption reduction in mitochondria, whereas anaerobic glycolysis initiates in the face of impaired utilisation of oxygen.^9^ As a transcription factor, HIF‐1α also activates the expression of glycolytic genes and promotes glycolysis.^10^, ^11^ It has been reported that glycolytic enzymes promote stiffness‐induced angiocrine signalling, resulting in inflammation and PHT and aggravating damage to the gastric mucosa.^12^ The roles of glycolysis, especially glycolytic metabolites, in regulating the machinery of gastric mucosal lesions in PHT and GC are still unclear.

The mitochondrion plays a central role in energy balance and associated redox systems for the regulation of the eukaryotic epigenome.^13^, ^14^ Stable mitochondrial dynamics mainly refer to balanced mitochondrial fusion and division to promote normal mitochondrial function. However, excessive mitochondrial fission may cause mitochondrial morphological alterations and dysfunction.^15^ Dynamin‐related protein 1 (Drp1) is a crucial regulator of mitochondrial fission and assembles into an oligomeric fission complex in the mitochondrial outer membrane.^16^ Notably, the rate‐limiting enzyme pyruvate kinase M2 (PKM2) in glycolysis interacts with key mitochondrial dynamics regulators to promote mitochondrial oxidative phosphorylation.^17^ To a lesser extent, adenosine triphosphate (ATP) synthesis by glycolysis is critical for triggering and maintaining mitochondrial fission activity.^18^ Mitochondrial fission can also shift glycolysis in different cell types.^19^ Mitochondrial fission accelerates inner membrane proton leakage and thus increases reactive oxygen species (ROS) levels, which causes increased oxidative stress and inflammation.^20^ Nevertheless, whether excessive glycolysis and mitochondrial dysfunction under hypoxia result in gastric mucosal lesion progression in PHT and GC has not yet been fully evaluated.

In the present study, we showed that HIF‐1α is upregulated in the gastric mucosa in PHT and GC, indicating the generality of hypoxia in gastric mucosal lesions. HIF‐1α activates METTL3‐mediated N6‐methyladenosine (m6A) methylation of Drp1 in an IGF2BP3‐dependent manner to enhance mitochondrial fission and dysfunction, and HIF‐1α also influences energy metabolism by enhancing lactate dehydrogenase A (LDHA)‐mediated glycolysis. Moreover, Drp1‐dependent mitochondrial fission and enhanced glycolysis are associated with alterations in antioxidant enzyme activity and dysfunction of the mitochondrial electron transport chain (ETC), resulting in massive mitochondrial ROS (mtROS) production, and this increase in mtROS activates the NLRP3 inflammasome to stimulate a large release of inflammatory mediators to form an anoxic and inflammatory microenvironment, which ultimately promotes the development of PHG and GC. Thus, hypoxia repair or targeting of the HIF‐1α‐related pathway may be effective treatments for these gastric mucosal diseases under hypoxic condition.

## 2 MATERIALS AND METHODS

### 2.1 Patient tissue samples

GC tissues were collected from six patients who underwent radical gastrectomy at the Third Affiliated Hospital of Sun Yat‐Sen University. Prior to surgery, none of the patients underwent chemotherapy or radiotherapy. The Endoscopic Center of the Third Affiliated Hospital of Sun Yat‐Sen University provided gastric mucosal specimens from 16 PHG patients (eight with hepatitis B virus‐infected liver cirrhosis and eight with portal vein occlusion [from the cavernous transformation of the portal vein or portal vein thrombosis without cirrhosis] without *Helicobacter pylori* infection who had not yet undergone any therapeutic intervention) and 16 uninvolved healthy volunteers who were undergoing regular healthy physical examination. Tanoue's three‐category scheme served as the foundation for the PHG classification.^21^ Prior to being used in the study, all tissues were obtained with the informed agreement of volunteers and patients, and the Hospital's Institute Research Ethics Committee approved the study procedure (no. RG2023‐032‐01).

### 2.2 Mouse models

Six‐week‐old *METTL3* wild‐type (*METTL3*‐WT) and *METTL3* mutant (*METTL3*‐Mut, strain no. T003080) mice and *NLRP3* wild‐type (*NLRP3*‐WT) and *NLRP3* knockout (*NLRP3*‐KO, strain no. T01087) mice on a C57BL/6 background were purchased from GemPharmatech. CRISPR/Cas9 technology was used to edit the *METTL3* gene in *METTL3*‐Mut mice. Briefly, the METTL3 gene has 15 transcripts. According to the structure of the *METTL3* gene, exon 2‒exon 3 of the METTL3‐201 (ENSMUST00000022767.15) transcript is recommended as the knockout region. First, the sgRNA was transcribed in vitro, and then the Cas9 and sgRNA were microinjected into the zygotes of C57BL/6 mice. F0‐positive mice were obtained from transplanted zygotes and confirmed by polymerase chain reaction (PCR) and sequencing. Finally, the F0‐positive generation mice were mated with C57BL/6 mice to obtain a stable F1 generation mouse model. In addition, the phenotype is available on the Mouse Genome Informatics website under the MGI number 1927165 (https://www.informatics.jax.org/marker/MGI:1927165). The mice used in the experiments were all 6‒8‐week‐old (18‒20 g) males, and they were then randomly divided into two groups: the portal vein ligation (PVL) model group and the sham operation (SO, without PVL) group. The PVL group was established using a PVL technique.^22^ Xenograft tumours in nude mice were established by subcutaneous injection of 5 × 10^6^ human GC cell line SGC7901 cells into both flanks of 6‐week‐old male BALB/c athymic nude mice. The data were recorded every 3 days after 1 week of inoculation. After 30 days of implantation, the tumour tissues were removed, and the tumour size and weight were calculated. The tumour volume was calculated according to the formula (.5 × length × width^2^). The Third Affiliated Hospital of Sun Yat‐Sen University and the Animal Care and Protection Committee of South China Agricultural University evaluated and authorised all experiments (no. 2022F122).

### 2.3 Drug treatment

For HIF‐1α inhibition, mice were administered PX‐478 (5 mg/kg, HY‐10231, MedChemExpress) in phosphate‐buffered saline (PBS) solution by oral gavage every other day, and the control mice received an equivalent amount of PBS. For NLRP3 inhibition, mice were gavaged with MCC950 (20 mg/kg, HY‐12815, MedChemExpress) or vehicle (.9% NaCl) every day for 5 days/week. For Drp1 inhibition, mice were given mitochondrial division inhibitor 1 (Mdivi‐1) (20 mg/kg, dissolved in dimethyl sulfoxide (DMSO), HY‐15886, MedChemExpress) intraperitoneally, and the control mice were injected with the same volume of DMSO. For ROS scavenging, mice were intraperitoneally injected with mito‐TEMPO (MT, dissolved in PBS, 10 mg/kg, HY‐112879, MedChemExpress) every other day, and the mice in the control group were given an equal volume of PBS intraperitoneally.

### 2.4 Sample collection

For histological analysis, stomach specimens from healthy volunteers, PHG patients and GC patients were fixed, and xenograft tumour mouse specimens were processed similarly. For PHT models, the stomach was harvested for histological analysis. The lesser curvature was longitudinally opened and mucosal layers were collected from the slides. The gastric injury indexes of the mouse models were analysed as previously described^23^: 0, normal; 1, mucosa with erosion; 2, mucosa with ulcers (<1 mm); 3, mucosa with ulcers (1‒2 mm); 4, mucosa with ulcers (3‒4 mm); and 5, mucosa with ulcers (>5 mm).

### 2.5 Histopathological staining

The harvested gastric mucosal tissues or GC tissues were fixed.^22^ The slides were incubated with the corresponding antibodies against METTL3 (ab195352, Abcam), HIF‐1α (sc‐13515, Santa Cruz), 4‐hydroxynonenal (4‐HNE, ab46545/ab48506, Abcam), Drp1 (8570S/ab156951, Cell Signalling Technology/Abcam), Ki67 (ab16667, Abcam), IGF2BP3 (14642‐1‐AP/ab177477, Proteintech/Abcam), LDHA (19987‐1‐AP, Proteintech) and NLRP3 (ab263899, Abcam). Immunofluorescence (IF) staining of the samples was performed using primary antibodies against Fis1 (A5821, ABclonal), Drp1 (8570S/ab156951, Cell Signalling Technology/Abcam), METTL3 (ab195352, Abcam), TOMM20 (ab186735/ab283317, Abcam), cytokeratin 18 (CK18, GTX105624, GeneTex), IGF2BP3 (14642‐1‐AP, Proteintech), NLRP3 (ab263899, Abcam) and interleukin (IL)‐1β (ab283818, Abcam). For double IF staining, a secondary targeted protein was examined after the initial protein detection stage. Nuclei were counterstained with 4′,6‐diamidino‐2‐phenylindole dihydrochloride (DAPI). Gastric sections were utilised for hypoxic status analysis with a Hypoxyprobe‐1 kit (Hypoxyprobe, Inc.). Transmission electron microscopy (TEM, Hitachi, H‐800) was used to examine gastric tissues or cell samples from a randomly selected pool of five fields. The length, width and area of the mitochondria were measured by ImageJ.

### 2.6 Primary cell isolation and experiments

GES‐1 cells (human gastric epithelial cell line) and SGC7901 cells were used. Primary gastric epithelial cells from the mouse models were isolated via collagenase perfusion as previously described^23^ and cultured. To observe the phenotype of the transient transfectants, Lipofectamine 2000 was used to knockdown *IGF2BP3* (*shIGF2BP3*, sc‐60846‐SH, Santa Cruz) and *METTL3* (*shMETTL3*, sc‐92172‐SH, Santa Cruz). To overexpress *HIF‐1α*, *HIF‐1α‐*Vector (as HIF‐1α) or vector (as Vector) plasmids were transfected into GES‐1 or SGC7901 cells.

### 2.7 Confocal microscopy

For Mito‐Tracker staining, cells treated with 400 nM Mito‐Tracker Red CM‐H2XRos (40740ES50, Yeasen) were fixed and permeabilised. Mitochondrial fragmentation and morphology were analysed by using ImageJ software. For IF staining, the cells were incubated with primary antibodies overnight at 4°C, followed by incubation with the appropriate secondary antibody labelled with Alexa 488 or Alexa 594, as well as DAPI. Finally, analyses were performed using a Zeiss LSM880/800 confocal microscope.

### 2.8 Western blotting and coimmunoprecipitation

The following antibodies were incubated: anti‐HIF‐1α (sc‐13515, Santa Cruz), anti‐Histone H3 (17168‐1‐AP, Proteintech), anti‐Drp1 (8570S/ab156951, Cell Signalling Technology/Abcam), anti‐Fis1 (A5821, ABclonal), anti‐Mff (17090‐1‐AP, Proteintech), anti‐Mid49 (28718‐1‐AP, Proteintch), anti‐Mid51 (20164‐1‐AP, Proteintch), anti‐mitofusin 1 (Mfn1; 13798‐1‐AP, Proteintech), anti‐mitofusin 2 (Mfn2; A12771, ABclonal), anti‐optic atrophy 1 (OPA1; A9833, ABclonal), anti‐4‐HNE (ab46545/ab48506, Abcam), anti‐Ki67 (ab16667, Abcam), anti‐METTL3 (ab195352, Abcam), anti‐IGF2BP3 (14642‐1‐AP, Proteintech), anti‐NLRP3 (ab263899, Abcam), anti‐hexokinase‐2 (HK2; ab209847, Abcam), anti‐PKM2 (A20991, ABclonal), anti‐LDHA (19987‐1‐AP, Proteintech), anti‐NADH ubiquinone oxidoreductase subunit A9 (NDUFA9; 20312‐1‐AP, Proteintech), anti‐succinate dehydrogenase complex flavoprotein subunit A (SDHA; 14865‐1‐AP, Proteintech), anti‐cytochrome b (Cyt b; 55090‐1‐AP, Proteintech), anti‐cyclooxygenase I (COX I; ab133319, Abcam), anti‐alpha subunit of ATP synthase (ATP5A; ab176569, Abcam), anti‐Cleaved caspase‐3 (ab32042, Abcam), anti‐Caspase‐1 (A0964/ab207802, ABclonal/Abcam), anti‐mixed lineage kinase domain‐like (MLKL, 66675‐1‐IG, Proteintch), anti‐p‐MLKL (ab196436, Abcam), anti‐IL‐1β (ab283818, Abcam), anti‐IL‐18 (10663‐1‐AP, Proteintech), anti‐GSDMD (ab219800/AF4012, Abcam/Affinity), anti‐ferritin heavy chain (FTH, ab75973, Abcam) and anti‐ferritin light chain (FTL, ab109373, Abcam) antibody. β‐Actin (sc‐47778, Santa Cruz) or Histone H3 (A2348, ABclonal, for nucleus) was used as an internal control. The results were analysed by ImageJ.^22^, ^24^ Coimmunoprecipitation was performed using a Kit (Thermo Scientific).^25^ The following antibodies (4 µg, as shown above) were used: Fis1 (A5821, ABclonal) and Drp1 (8570S/ab156951, Cell Signalling Technology/Abcam).

### 2.9 ATP measurement

Gastric tissues were collected according to previous methods, and ATP levels in gastric tissue sections were measured using a kit (Beyotime Biotechnology). Twenty microlitres of the supernatant was mixed with 100 µL of luciferase reagent, and the ATP concentrations in the tissues were measured by the luminescence of each sample via microtitre plate photometry. The ATP level is based on a standard curve of ATP.

### 2.10 Luciferase reporter gene assay

The target promoters *METTL3* or *LDHA* were inserted in front of the luciferase reporter gene to construct the reporter plasmid. The plasmids that could overexpress HIF‐1α (plasmid *pcDNA3.1‐HIF‐1α‐*Vector [as *HIF‐1α‐*Vector] or *pcDNA3.1‐*vector [as Vector]) were cotransfected with the reporter plasmid into GES‐1 or SGC7901 cells. Luciferase activity was measured with a kit (RG028, Beyotime Biotechnology).^25^

### 2.11 Energy metabolism analysis

Energy metabolism analysis was performed by Shanghai Metabo‐Profile Corporation. Energy metabolism in isolated primary gastric epithelial cells was detected and analysed via a targeted metabolism technology platform (ultra performance liquid chromatography‐triple quadrupole mass spectrometry, UPLC‐MS/MS). The raw data were further processed using MassLynx (V4.1, Waters) and iMAP software (V1.0, Metabo‐type Spectrum Biotechnology).

### 2.12 Microarray analysis

Total RNA was extracted, purified and amplified to generate Cy3dCTP‐labelled cDNA, and the purified double‐stranded cDNA product was eluted according to the instructions and evaporated under vacuum. Afterward, the transcription reaction was carried out with a T7 enzyme mixture at 37°C for 14 h.^25^ An Agilent hybridisation oven was used to complete array hybridisation overnight. The data were log2 transformed and hierarchically clustered using the Adjust Data function of the CLUSTER 3.0 software. Tree visualisation was done using Java TreeView (Stanford University School of Medicine).^23^

### 2.13 Methylated RNA immunoprecipitation sequencing

Methylated RNA immunoprecipitation sequencing (MeRIP‐seq) was performed using a kit (NEB) from Guangzhou Huayin Medical Laboratory Center. Briefly, the fragmented RNA was incubated with a m6A antibody and immunoprecipitated (NEB). mRNAs containing m6A were enriched and subjected to high‐throughput sequencing and validation.

### 2.14 Separation of the cytoplasm and mitochondria

The mitochondria from the gastric mucosal epithelial cells were lysed on ice with Cellular Mitochondrial Isolation Buffer according to the instructions of the Cellular Mitochondrial Isolation Kit (ab110170, Abcam). The mitochondria and cytoplasm were centrifuged (600 × *g*, 15 min at 4°C, then 11 000 × *g*, 10 min at 4°C). The pellet (the mitochondrial fraction) was collected and resuspended in mitochondrial lysis buffer for further analysis. Subsequently, the cytosolic fraction was obtained at 12 000 × *g* (15 min, 4°C).

### 2.15 Determination of oxidative stress

The ROS levels were measured with a kit (Beyotime Biotechnology). 2,7‐Dichlorodihydrofluorescein diacetate was used to incubate the cells and then examined by fluorescence spectrophotometer. Malondialdehyde (MDA) levels were measured by an MDA assay kit (Abcam, ab118970). MitoSox (Thermo Fisher) was observed with a fluorescence microscope (Imager Z2, Zeiss). The cells were incubated with 1.0 mL of 5 µM MitoSox. Hoechst 33258 was used to stain the nuclei.

JC‐1 fluorescence test kit (Beyotime Biotechnology) was used to test the mitochondrial membrane potential (ΔΨm). For the green JC‐1 monomers, images were examined at 490 nm excitation and 530 nm emission, and for the red JC‐1 aggregates, images were examined at 540 nm excitation and 590 nm emission.

In order to measure oxidised mitochondrial DNA (ox‐mtDNA), mtDNA was purified using an AllPrep DNA/RNA Mini Kit (Qiagen). Next, the 8‐hydroxy‐2'‐deoxyguanosine (8‐OHdG) content of the mtDNA (mt 8‐OHdG) was measured (Cell Biolabs).

### 2.16 Ca^2+^ concentration detection and enzyme‐linked immunosorbent assay

The intracellular Ca^2+^ concentration ([Ca^2+^]_i_) was measured by Fluo‐3AM (a fluorescent Ca^2+^ indicator, ab145254, Abcam) and the mitochondrial Ca^2+^ concentration ([Ca^2+^]_m_) was examined by Rhod‐2AM (a fluorescent Ca^2+^ indicator, ab142780, Abcam). The fluorescence signals were detected with a Leica STELLARIS STED confocal microscope. The concentrations of IL‐1β and IL‐18 were measured (R&D Systems).

### 2.17 Flow cytometry analysis

Fluorochrome‐labelled antibodies were utilised. After labelling the cells with an anti‐NLRP3 antibody (ab263899, Abcam), PBS was washed and FlowJo V.X software was utilised to examine the fluorescence, and data were obtained using a BD LSRFortessa cytometer.

### 2.18 Seahorse assay

To overexpress HIF‐1α, GES‐1 cells were transfected with *HIF‐1α‐*Vector (HIF‐1α) or vector (Vector) plasmids (RiboBio Co). The transfection medium was replaced with regular culture medium after 1 day of incubation. For Drp1 inhibition, GES‐1 cells and SGC7901 cells were treated with 10 µM Mdivi‐1 (HY‐15886, MedChemExpress) after transfection. We measured mitochondrial respiration (oxygen consumption rate, OCR) and the glycolytic rate (extracellular acidification rate, ECAR) by using the Cell Mito Stress Test Kit (103015‐100, Agilent) and the Glycolytic Rate Assay Test Kit (103710‐100, Agilent), respectively. The OCR is a key parameter for characterising mitochondrial function, and its values were determined by a Seahorse XF96 extracellular flux analyser. We added 1.5 µmol/L oligomycin, 2 µmol/L carbonyl cyanide‐4 (trifluoromethoxy) phenylhydrazone and .5 µmol/L rotenone/antimycin A (Rot/AA) to the culture plate. Finally, the maximal respiration and nonmitochondrial respiration values were recorded. For the glycolytic rate test, after the baseline ECAR was measured, .5 µmol/L Rot/AA and 50 mmol/L 2‐deoxyglucose were added sequentially. The glycolytic levels of the cells were determined by the glycoPER value. After treatment, Dulbecco's Modified Eagle Medium (DMEM, pH 7.4, 103575‐100, Agilent) was preheated in a 37°C water bath. The culture medium (pH 7.4) contained glucose, pyruvate and glutamine, and the cells were subsequently incubated without CO_2_ (1 h, 37°C) and finally tested on a Seahorse XF96 extracellular flux analyser.

### 2.19 RNA immunoprecipitation (RIP) and chromatin immunoprecipitation (ChIP) assays

The assay was performed using a kit (Geneseed, P0101). Briefly, protein A/G magnetic beads and cell lysates supplemented with RNase inhibitor were incubated with anti‐IGF2BP3 (Abcam, ab177477) and anti‐IgG throughout the night. Then, the RNA‒protein complexes were washed and proteinase K digestion buffer was added to remove the proteins. Finally, RNA was extracted for qPCR analysis. The relative enrichment was normalised to the input as follows: %Input = 1/10 × 2^Ct[IP] ‒ Ct[input]^. Agarose electrophoresis assay for quantifying RNA‒protein interactions. ChIP assay was performed following the instruction of the kit (Cell signaling Technology). The ChIP‐enriched DNA was applied for real‐time PCR assay, and the data were normalized to the values of the same input sample.

### 2.20 Public data analysis

Gene expression profile data comparing GC tissues (*n* = 300) to normal gastric tissues (*n* = 100) were provided by the GEO (https://www.ncbi.nlm.nih.gov/geo/). After excluding samples without clinical information, analysis of the GSE66229 dataset revealed METTL3 levels in normal and tumour tissues. RNA sequencing (RNA‐seq) data from 375 gastric tumour sections and 32 gastric normal tissue samples were also acquired from The Cancer Genome Atlas (TCGA) (https://portal.gdc.cancer.gov). The difference in METTL3 expression between tumour and normal tissues was determined with GraphPad Prism.

### 2.21 Statistical analysis

All the experiments were repeated at least three times, and different representative specimens are presented for the related images or bands in the current experiments. The results are expressed as the means ± standard errors of the means. Student's two‐tailed paired *t*‐test, one‐way analysis of variance (ANOVA) (more than two groups of data, single factor) or two‐way ANOVA (more than two groups of data, two factors) with repeated measures followed by Bonferroni's comparison post hoc test, and post hoc tests were run only if *F* achieved *p *< .05 were carried out. *p *< .05 was considered to indicate statistical significance.

## 3 RESULTS

### 3.1 HIF‐1α is involved in mitochondrial dysfunction under hypoxia in both PHG and GC

**FIGURE 1:**
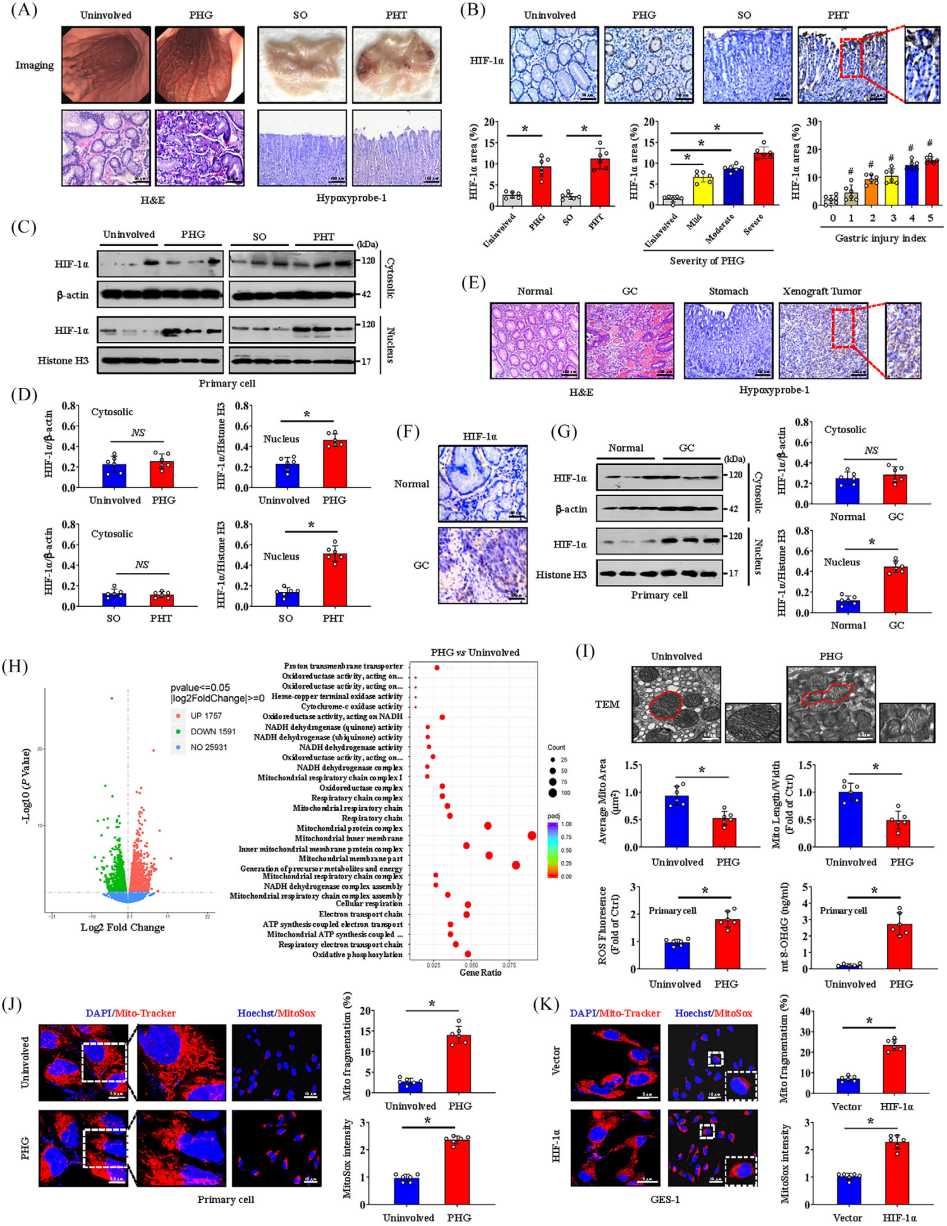
Hypoxia‐inducible factor‐1α (HIF‐1α) is involved in mitochondrial dysfunction under hypoxia in both portal hypertensive gastropathy (PHG) and gastric cancer (GC). (A) Panel on the left: representative gastric endoscopic image and haematoxylin and eosin (H&E) staining of the gastric mucosa of PHG patients compared to that of uninvolved healthy individuals. Right panel: gross image and Hypoxyprobe‐1 immunohistochemical (IHC) staining (brown) of the gastric mucosa in portal hypertension (PHT) induced by portal vein ligation (PVL) compared to that in sham operation (SO). *n* = 6 per group. (B) HIF‐1α IHC staining (brown) and HIF‐1α area (%) were presented. The percentage of the HIF‐1α‐positive area corresponding to PHG severity and the gastric injury index of PHT was also analysed. *n* = 6 per group. ^*^ *p* < .05, ^#^ *p* < .05 versus the group with a gastric injury index of 0. (C) Western blotting showing HIF‐1α expression in the cytosol and nucleus in the indicated groups. (D) The densitometry units of HIF‐1α/β‐actin and HIF‐1α/Histone3 from (C). *n* = 6 per group, ^*^ *p* < .05, NS: not significant. (E) Images of H&E and Hypoxyprobe‐1 staining (brown) of the indicated gastric mucosa sections. *n* = 6 per group. (F) Representative images of HIF‐1α IHC staining (brown) of normal and GC tissues. (G) Immunoblot analyses of HIF‐1α in the cytosol and nucleus of normal and GC tissues. The ratios of densitometry units of HIF‐1α/β‐actin and HIF‐1α/Histone H3 were analysed. *n* = 6 per group. ^*^ *p* < .05, NS: not significant. (H) Volcano plot of differentially expressed genes in the PHG groups compared with those in the uninvolved groups. Kyoto Encyclopedia of Genes and Genomes (KEGG) pathway enrichment of signalling pathways according to the RNA sequencing assay. *n* = 3 in each group. (I) Ultrastructural features identified by transmission electron microscopy (TEM) in the uninvolved and PHG groups. The quantified mitochondrial area and the ratio of mitochondrial length to width were measured. The levels of reactive oxygen species (ROS) and mitochondrial 8‐OHdG (mt 8‐OHdG) in primary gastric epithelial cells were shown (lower panel). *n* = 6 per group. ^*^ *p* < .05. (J and K) Mito‐Tracker (red) and MitoSox (red) staining of the indicated groups. Nuclei (blue) were counterstained with 4′,6‐diamidino‐2‐phenylindole dihydrochloride (DAPI) or Hoechst. The histogram presented the ratio of mitochondrial fragmentation and MitoSox intensity. *n* = 6 per group. ^*^ *p* < .05.

To evaluate the hypoxic environment of the gastric mucosa in PHT, gastric mucosal tissues from patients clinically diagnosed with PHG were collected, and a PHT mouse model was established. The results showed that PHT caused extensive erythema, hyperaemia, oedema and inflammatory infiltration of the gastric mucosa (Figure 1A). Moreover, positive signals of hypoxyprobe‐1 indicated hypoxia in the gastric mucosa of PHT mice (Figure 1A). Further immunohistochemical staining indicated that HIF‐1α was elevated in both human and mouse portal hypertensive gastric mucosal tissues and was positively correlated with the severity of PHG and the gastric injury index of PHT mice (Figure 1B). As a transcription factor, the increased expression of HIF‐1α in the gastric mucosa was mainly concentrated in the nucleus rather than in the cytoplasm (Figure 1C,D). In addition to identifying benign gastric disease, to further determine the hypoxic condition in malignant gastric mucosal lesions, we collected clinical GC tissues and established a SGC7901 mouse gastric graft tumour model, and the related results revealed similar changes in hypoxia and localised nuclear expression of HIF‐1α in cancerous tissues but not in normal gastric mucosa (Figure 1E‒G). Transcriptomic sequencing of human normal and PHG patients tissues revealed that the expression of many genes was aberrant under PHT and that mitochondrial dysfunction‐ and oxidative stress‐related pathways were enriched (Figure 1H). TEM revealed mitochondrial damage and morphological changes, and ROS and 8‐OHdG assays in primary gastric mucosal epithelial cells suggested that PHT enhanced mitochondrial oxidative stress (Figure 1I). Moreover, Mito‐Tracker and MitoSox probes showed an increase in mitochondrial network structure alterations and oxidative stress activation in primary cells from portal hypertensive gastric mucosa (Figure 1J). Alterations in mitochondrial network structure and MitoSox signalling were also observed in primary GC cells (Figure S1A). HIF‐1α overexpression in GES‐1 cells also induced abnormal mitochondrial morphology, decreased membrane potential, disturbed mitochondrial Ca^2+^ homeostasis and enhanced oxidative stress, suggesting changes in mitochondrial dysfunction (Figures 1K and S1B,C). In addition, MitoSox detection revealed that increased oxidative stress in cancerous tissues from SGC7901 mouse gastric graft tumour models (Figure S1D). Therefore, HIF‐1α is involved in the mitochondrial dysfunction of gastric mucosal lesions in PHT and GC under hypoxia.

### 3.2 HIF‐1α mediates mitochondrial dysfunction via Drp1‐dependent mitochondrial fission

**FIGURE 2:**
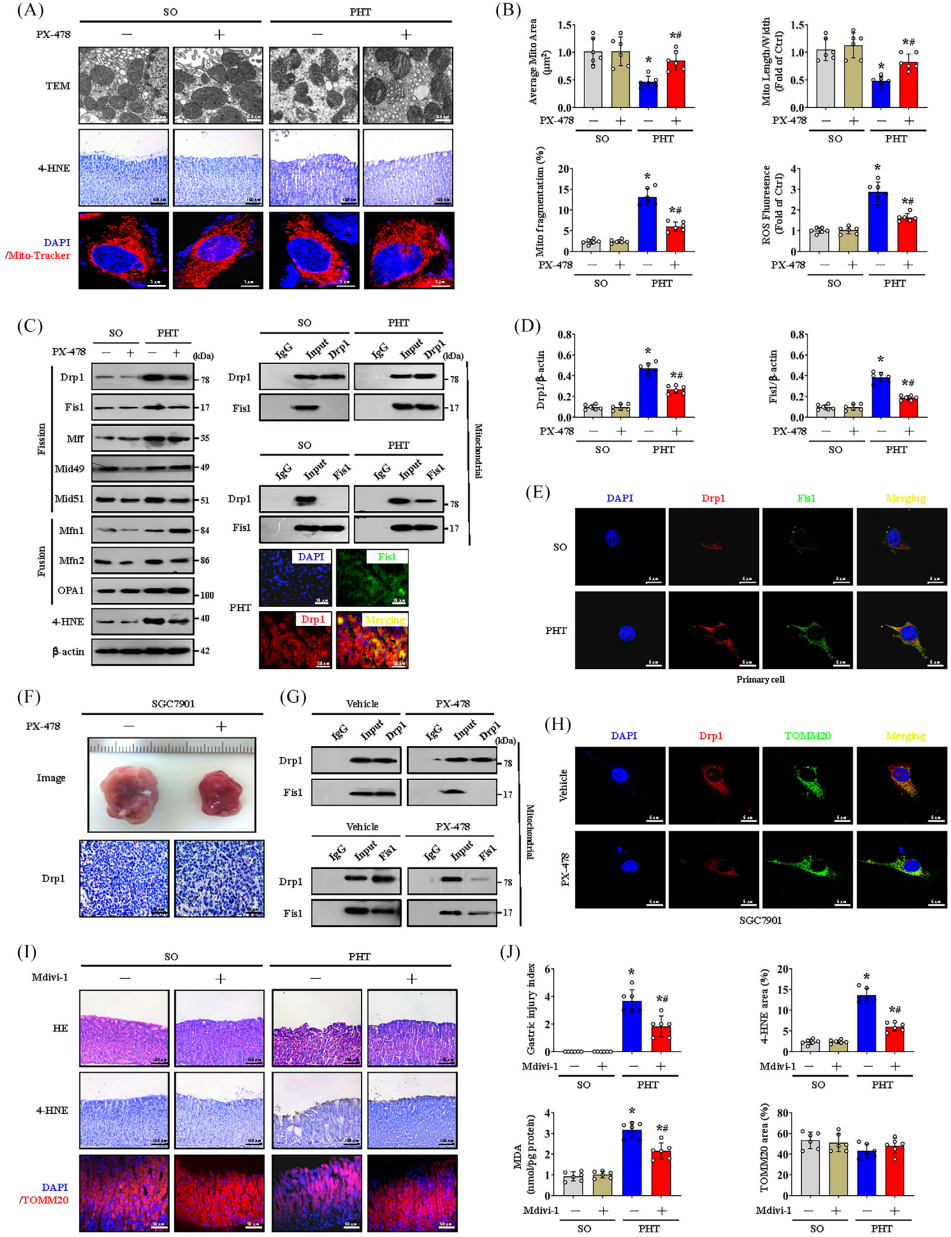
Hypoxia‐inducible factor‐1α (HIF‐1α) mediates mitochondrial dysfunction via dynamin‐related protein 1 (Drp1)‐dependent mitochondrial fission. (A) Transmission electron microscopy (TEM), 4‐hydroxynonenal (4‐HNE) (brown) and Mito‐Tracker (red) analyses of the indicated gastric sections or primary cells from the sham operation (SO) and portal hypertension (PHT) groups that received or those did not receive PX‐478 treatment. (B) The quantified mitochondrial area and the ratio of mitochondrial length to width were measured by TEM. The degree of mitochondrial fragmentation and the levels of reactive oxygen species (ROS) were also analysed. *n* = 6 per group. ^*^ *p* < .05 versus SO mice, ^#^ *p* < .05 versus PHT mice without PX‐478 treatment. (C) Immunoblot analysis of mitochondrial fission and fusion proteins. Coimmunoprecipitation (Co‐IP) and double immunofluorescence (IF) staining analyses of the interaction between Drp1 and Fis1. *n* = 6 per group. (D) The histogram showed the densitometry units of Drp1/β‐actin and Fis1/β‐actin from (C). *n* = 6 per group. ^*^ *p* < .05 versus SO mice, ^#^ *p* < .05 versus PHT mice without PX‐478 treatment. (E) Co‐staining for Drp1 (red) and Fis1 (green) in isolated primary cells under a confocal microscope. Nuclei (blue) were counterstained with 4′,6‐diamidino‐2‐phenylindole dihydrochloride (DAPI). (F) Gross appearance and Drp1 staining of gastric tumour tissues from the indicated groups treated with or without PX‐478. (G) Interactions between Drp1 and Fis1 in mitochondria were analysed by Co‐IP. (H) Co‐staining of Drp1 (red) and TOMM20 (green) was shown, and nuclei (blue) were counterstained with DAPI. (I) Haematoxylin and eosin (H&E), 4‐HNE (brown) and TOMM20 (red) staining of gastric mucosal tissues from the indicated mouse models. (J) The gastric injury index, 4‐HNE area (%), malondialdehyde (MDA) levels and TOMM20 area (%) from (I) were presented. *n* = 6 per group. ^*^ *p* < .05 versus SO mice, ^#^ *p* < .05 versus PHT mice without mitochondrial division inhibitor 1 (Mdivi‐1) treatment.

To further determine the role of hypoxia and HIF‐1α in gastric mucosal lesions, PX‐478 (a HIF‐1α inhibitor) was used. TEM analysis and 4‐HNE staining demonstrated that PX‐478 alleviated PHT‐mediated mitochondrial dysfunction and oxidative stress in the gastric mucosa (Figure 2A). Mito‐Tracker fluorescence of primary gastric mucosal epithelial cells also revealed changes in mitochondrial morphology caused by PHT, and PX‐478 reversed these changes and decreased ROS production (Figure 2A,B). Mitochondrial fusion and fission are essential for stable mitochondrial function. Subsequently, we found that the mitochondrial division‐driven proteins Drp1 and Fis1 were significantly upregulated in PHT‐induced gastric mucosal injury and decreased in response to PX‐478 (Figure 2C,D). In gastric tissues and primary gastric mucosal epithelial cells from the PHT‐induced model, double IF staining showed increased colocalisation of Drp1 and Fis1, and IP further highlighted that this interaction was concentrated in mitochondria (Figure 2C,E). Drp1 expression (Drp1 area: 6.17% in normal tissues vs. 26.50% in GC tissues) was also increased in GC tissues and was associated with enhanced cell proliferation (Ki67 index: 12.50% in normal tissues vs. 43.33% in GC tissues) (Figure S2A,B). In the established gastric tumour mouse models, we found that PX‐478 reduced tumour size (average tumour size: 14.65 mm in SGC7901 xenograft mouse models without PX‐478 vs. 8.45 mm in those with PX‐478) and tumour weight (5.25% in SGC7901 xenograft mouse models without PX‐478 vs. 2.82% in those with PX‐478) (Figures 2F and S2C), and PX‐478 repressed the expression of Drp1 and blocked the interaction between Drp1 and Fis1 in mitochondria (Figures 2G–H). The recruitment of Drp1 by adaptors at mitochondrial constriction sites controls the mitochondrial fission. Drp1 oligomerisation followed by mitochondrial constriction increases mitochondrial fission.^26^ Fis1 oligomers can promote oligomerisation of the mitochondrial adaptor Mid51 to drive Drp1 oligomerisation in mitochondria.^27^ Drp1 oligomerisation can regulate its binding to Mid49, Mid51 and Mff. Moreover, Mff oligomerisation is needed in cells for Drp1 recruitment and mitochondrial fission.^28^ Based on the important role of protein oligomerisation in mitochondrial fission, we also analysed the oligomeric state of Drp1, Fis1, Mff, Mid49, Mid51, Mfn1, Mfn2 and OPA1 in gastric mucosal lesions in PHT and found that PHT affected the oligomeric state of Drp1 and Fis1 in gastric epithelial cells from the mouse model, facilitating Drp1 and Fis1 oligomerisation (Figure S3A). Moreover, the oligomerisation of Mff, Mid49 and Mid51 in the epithelial cells of the PHT mouse model was slightly increased rather than that of Mfn1, Mfn2 and OPA1 (Figure S3A). We also found that the oligomeric states of Drp1 and Fis1 were affected in cells isolated from SGC7901 xenograft mice and decreased by PX‐478 treatment via utilising SGC7901 xenograft mouse models (Figure S3B). Although oligomerisation of Mff, Mid49, Mid51, Mfn1 and Mfn2 was also observed in cells isolated from SGC7901 xenograft mice, their oligomeric states were not significantly influenced by PX‐478 treatment (Figure S3B). These findings revealed that enhanced Drp1 and Fis1 oligomerisation contributes to HIF‐1α‐regulated mitochondrial fission in both PHT and GC.

Then, we used Mdivi‐1 (an inhibitor of Drp1) to clarify the effect of Drp1‐mediated mitochondrial fission and found that Mdivi‐1 alleviated gastric mucosal injury and oxidative stress (as indicated by the MDA and 4‐HNE levels) in PHT‐mediated gastric mucosa, although TOMM20 analysis revealed no significant change in the total mitochondrial area (Figure 2I,J). Moreover, Mdivi‐1 also alleviated GC cell proliferation (Ki67 index: 40.50% in SGC7901 xenograft mouse models without Mdivi‐1 vs. 20.17% in those with Mdivi‐1) and oxidative stress (4‐HNE area: 18.67% in SGC7901 xenograft mouse models without Mdivi‐1 vs. 10.00% in those with Mdivi‐1) in SGC7901 mouse models (Figure S2D). In summary, HIF‐1α inhibition alleviates mitochondrial oxidative stress and dysfunction by alleviating Drp1‐mediated mitochondrial fission, which can relieve the development of PHG and GC.

### 3.3 Drp1 expression is regulated by METTL3‐mediated m6A modification

**FIGURE 3:**
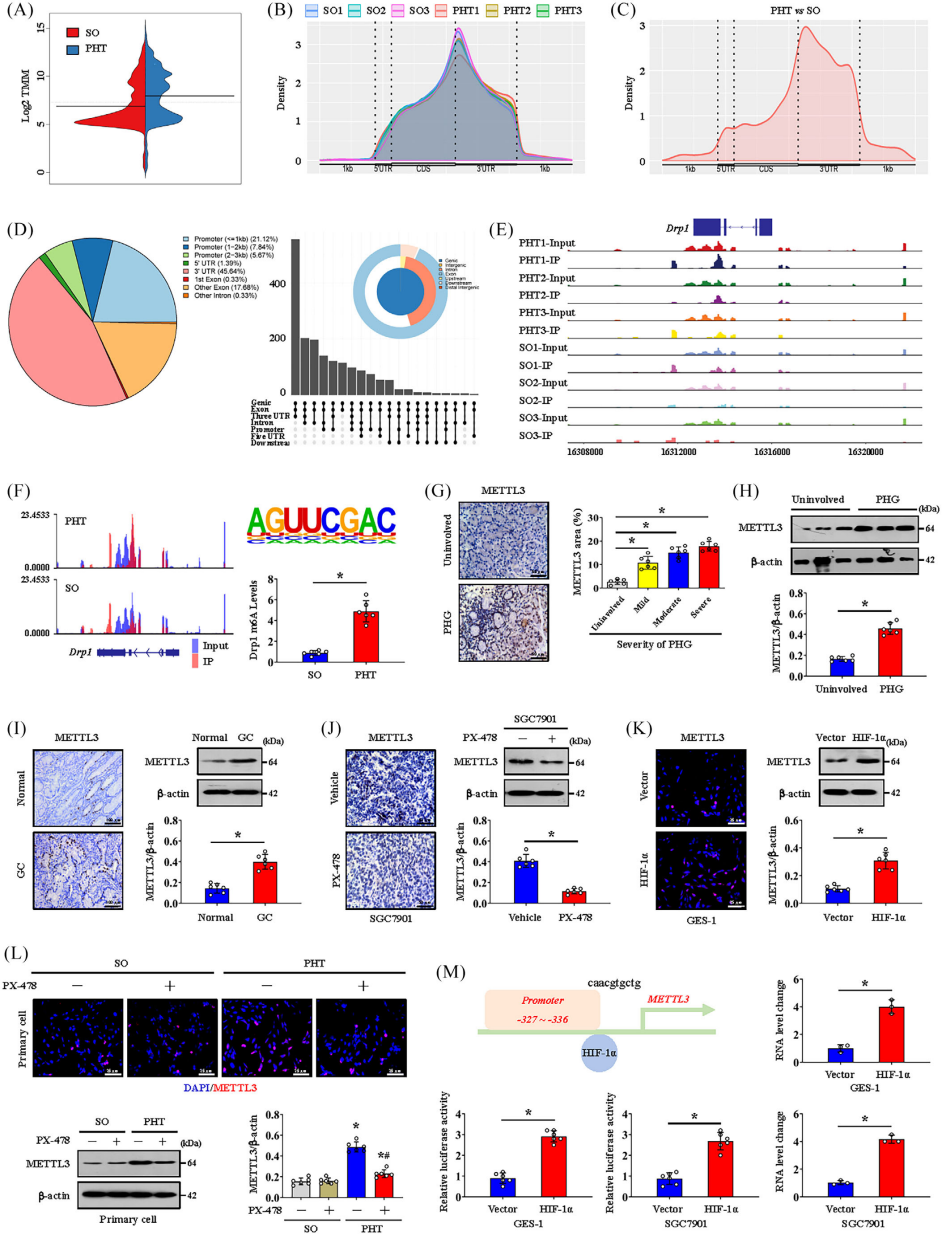
Dynamin‐related protein 1 (Drp1) expression is regulated by METTL3‐mediated N6‐methyladenosine (m6A) modification. (A) A beanplot of the distribution of specific numbers of peak‐bound mRNAs. (B) The peak regions of the distribution of relative gene positions of peaks in different groups. (C) The peak regions of the distribution of relative gene positions of the differential peak (portal hypertension [PHT] vs. sham operation [SO]). (D) The distribution of peaks on gene functional elements according to pie and UpSet plot analyses. (E) The signal visualisation of the differential peak regions of *Drp1*. (F) The m6A abundances in *Drp1* transcripts in PHT groups relative to those in SO groups (left panel). The m6A consensus sequence motif was identified (right panel), and the Drp1 m6A levels in SO and PHT were also analysed. ^*^ *p* < .05. (G) METTL3 staining (brown) in the indicated groups was shown. The expression of METTL3 was related to the severity of portal hypertensive gastropathy (PHG). *n* = 6 per group. ^*^ *p* < .05. (H) The protein levels of METTL3 were analysed by western blotting. The ratio of densitometry units of normalised METTL3/β‐actin was further determined. *n* = 6 per group. ^*^ *p *< .05. (I and J) IHC staining (brown) and western blotting of METTL3 in the indicated groups. The ratio of densitometry units of normalised METTL3/β‐actin was further determined. *n* = 6 per group. ^*^ *p *< .05. (K) METTL3 immunofluorescence (IF) staining (red) and western blotting analyses of vector‐ and *HIF‐1α*‐transfected GES‐1 cells. The ratio of densitometry units of normalised METTL3/β‐actin was further determined. *n* = 6 per group. ^*^ *p *< .05. (L) IF (red) and immunoblot detection of METTL3 in the SO and PHT groups with or without PX‐478 treatment. The ratio of densitometry units of normalised METTL3/β‐actin was further determined. *n* = 6 per group. ^*^ *p* < .05 versus SO mice, ^#^ *p* < .05 versus PHT mice without PX‐478 treatment. (M) The binding sequence of hypoxia‐inducible factor‐1α (HIF‐1α) to the *METTL3* promoter was shown. The *METTL3* luciferase reporter activities in GES‐1 or SGC7901 cells transfected with vector or *HIF‐1α* were shown. *n* = 6 per group. ^*^ *p *< .05. The HIF‐1α‐associated complex was enriched in a specific region of the *METTL3* promoter, as shown by ChIP analyses. *n* = 3 per group. ^*^ *p *< .05. DAPI, 4′,6‐diamidino‐2‐phenylindole dihydrochloride; GC, gastric cancer.

m6A methylation plays important roles in various diseases. To understand the effects of m6A modification on PHT‐related gastric mucosal injury, mouse gastric mucosal tissues from the SO and PVL groups were isolated for MeRIP‐seq analysis. The data indicated that the m6A level in the PHT group was obviously different from that in the SO group (Figure 3A), and the distributions of the methylated peaks and differentially modified genes were mainly close to the 3′UTRs of the RNA structures and gene functional elements (Figure 3B–D). The m6A level of Drp1 was 5.6‐fold higher in the PHT group compared with that in the SO group, and the m6A consensus sequence AGUUCGAC motif was enriched within m6A sites in the immunopurified RNA, indicating the importance of Drp1 m6A modification in portal hypertensive gastric mucosal injury (Figure 3E,F). The expression of the methyltransferase METTL3, the key ‘writer’ of m6A modification, which is closely related to the severity of PHG, was increased in damaged gastric mucosal tissues in PHG (Figure 3G,H). Moreover, upregulated METTL3 was also confirmed in GC tissues (Figure 3I). The expression of *METTL3* mRNA was significantly higher in GC tissues than in normal tissues in the published clinical datasets TCGA and GSE66229 (Figure S4A). Furthermore, we found that PX‐478 suppressed METTL3 expression in mouse gastric graft tumour models and in the gastric mucosa of mice with PHT (Figure 3J,L). HIF‐1α overexpression in GES‐1 cells also promoted METTL3 expression (Figure 3K). HIF‐1α was predicted to bind to the *METTL3* promoter to activate its transcription (Figure 3M), and dual‐luciferase assays in GES‐1 and SGC7901 cells revealed that the *METTL3* reporter was activated by HIF‐1α. ChIP assays further showed that HIF‐1α could directly bind to the *METTL3* promoter (Figure 3M). In this study, METTL3‐mediated m6A methylation of Drp1 is involved in the pathogenesis of gastric mucosal lesions under HIF‐1α‐related hypoxic condition.

### 3.4 METTL3 mutation rescues mitochondrial function by inhibiting Drp1‐induced mitochondrial fission

**FIGURE 4:**
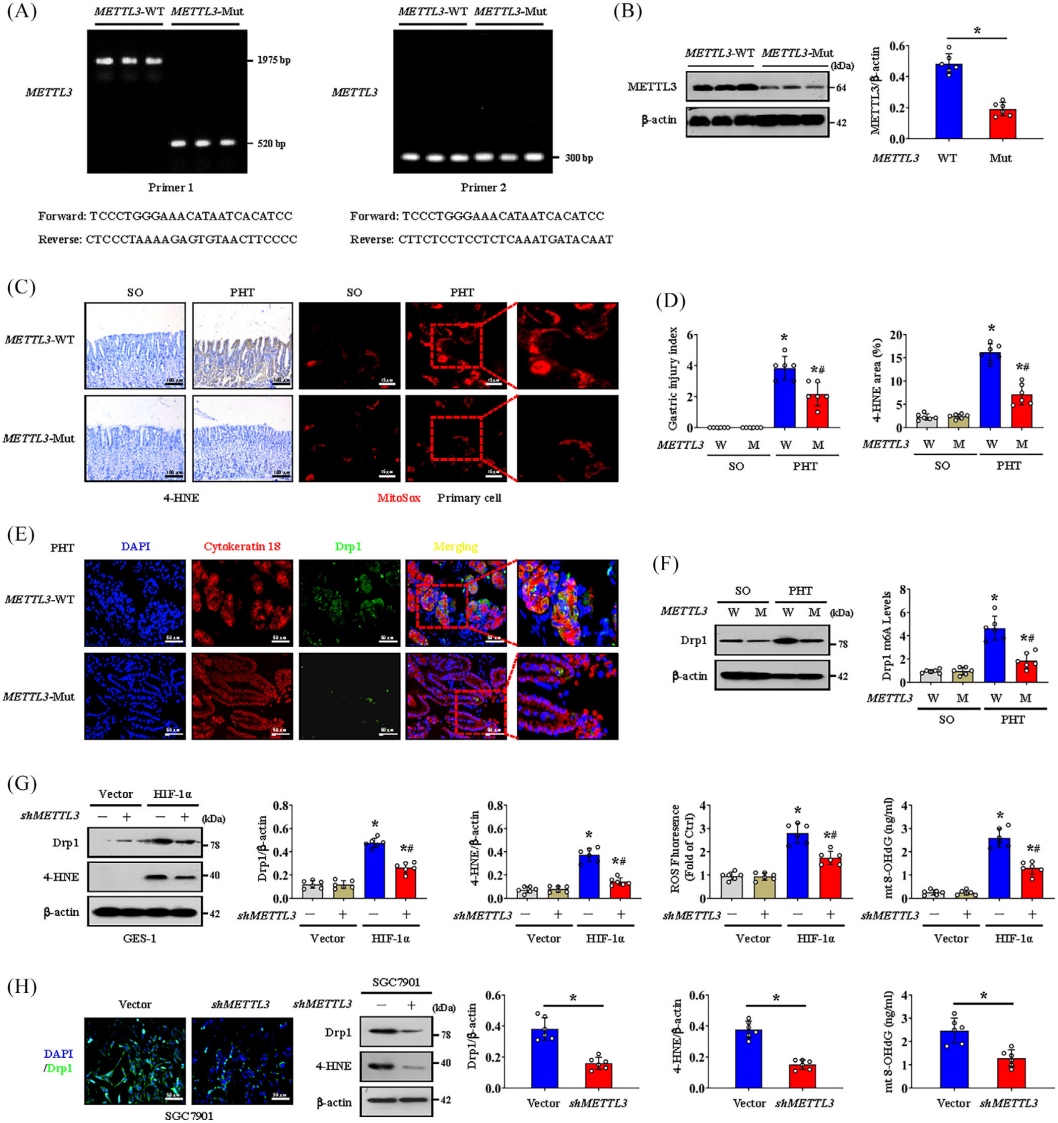
*METTL3* mutation rescues mitochondrial function by inhibiting dynamin‐related protein 1 (Drp1)‐induced mitochondrial fission. (A) Genotyping of *METTL3* wild‐type (*METTL3*‐WT) and *METTL3* mutant (*METTL3*‐Mut) mice was performed by using *METTL3* allele‐specific primers to analyse genomic DNA from tail snips. (B) The protein levels of METTL3 in the gastric mucosa of untreated *METTL3*‐WT and *METTL3*‐Mut mice were measured by western blotting. *n* = 6 per group. ^*^ *p* < .05. (C) Images of 4‐hydroxynonenal (4‐HNE) (brown) and MitoSox (red) staining in *METTL3*‐WT and *METTL3*‐Mut mice. (D) The gastric injury index and the 4‐HNE area were analysed in *METTL3*‐WT (W) and *METTL3*‐Mut (M) mice. ^*^ *p* < .05 versus sham operation (SO) mice, ^#^ *p* < .05 versus portal hypertension (PHT) *METTL3*‐WT mice. (E) Double immunofluorescence (IF) staining of Drp1 (green) and cytokeratin 18 (red) with 4′,6‐diamidino‐2‐phenylindole dihydrochloride (DAPI) (blue) counterstaining for DNA in gastric sections of the PHT‐induced *METTL3*‐WT or *METTL3*‐Mut mouse model. (F) The levels of Drp1 in *METTL3*‐WT (W) or *METTL3*‐Mut (M) mice were measured by western blotting. Drp1 m6A levels were further analysed. *n* = 6 per group. ^*^ *p* < .05 versus SO mice, ^#^ *p* < .05 versus PHT *METTL3*‐WT mice. (G) The protein levels of Drp1 and 4‐HNE in vector‐ and *HIF‐1α*‐transfected GES‐1 cells were measured by western blotting. The ratio of densitometry units of normalised Drp1/β‐actin and 4‐HNE/β‐actin and the levels of reactive oxygen species (ROS) and mitochondrial 8‐OHdG (mt 8‐OHdG) were further determined. *n* = 6 per group. ^*^ *p* < .05 versus the vector group, ^#^ *p* < .05 versus hypoxia‐inducible factor‐1α (HIF‐1α)‐transfected cells without *METTL3* knockdown (*shMETTL3*). (H) IF staining (green) of Drp1 and immunoblot analyses of Drp1 and 4‐HNE in the indicated groups of SGC7901 cells with or without *METTL3* knockdown. The ratio of densitometry units of normalised Drp1/β‐actin and 4‐HNE/β‐actin and the levels of mt 8‐OHdG were further examined. *n* = 6 per group. ^*^ *p* < .05.

*METTL3*‐Mut mice were further constructed to investigate the effects of METTL3‐mediated m6A modification of Drp1 on mitochondrial dysfunction and gastric mucosal lesions. Genotyping of *METTL3*‐WT and *METTL3*‐Mut mice was performed by analysing genomic DNA from tail snips (Figure 4A), and METTL3 expression in the gastric mucosa of *METTL3*‐Mut mice was lower than that in the gastric mucosa of *METTL3*‐WT mice (Figure 4B). MitoSox probing and 4‐HNE detection revealed that *METTL3* mutation reduced PHT‐induced mitochondrial oxidative stress and gastric injury in the gastric mucosa (Figure 4C,D). As a consequence, the localised expression of Drp1 in the gastric mucosal epithelial cells from PHT mice was decreased by *METTL3* mutation (Figure 4E). Consistently, the Drp1 m6A level and protein expression were inhibited in the *METTL3* mutational group after PHT modelling (Figure 4F). In vitro, HIF‐1α‐induced changes in Drp1 and 4‐HNE were reversed by *METTL3* knockdown (*shMETTL3* transfection) in GES‐1 cells, accompanied by decreased mitochondrial oxidative stress and ROS production (as detected by 4‐HNE, ROS levels and mt 8‐OHdG) (Figure 4G). Moreover, by analysing GC cells isolated from SGC7901 mouse gastric graft tumour models, we also found that *METTL3* knockdown had a similar effect (Figure 4H). Hence, *METTL3* mutation inhibits Drp1 m6A modification and expression, which can alleviate Drp1‐induced mitochondrial fission to rescue mitochondrial function.

### 3.5 METTL3 regulates Drp1 m6A modification and expression in an IGF2BP3‐dependent manner

**FIGURE 5:**
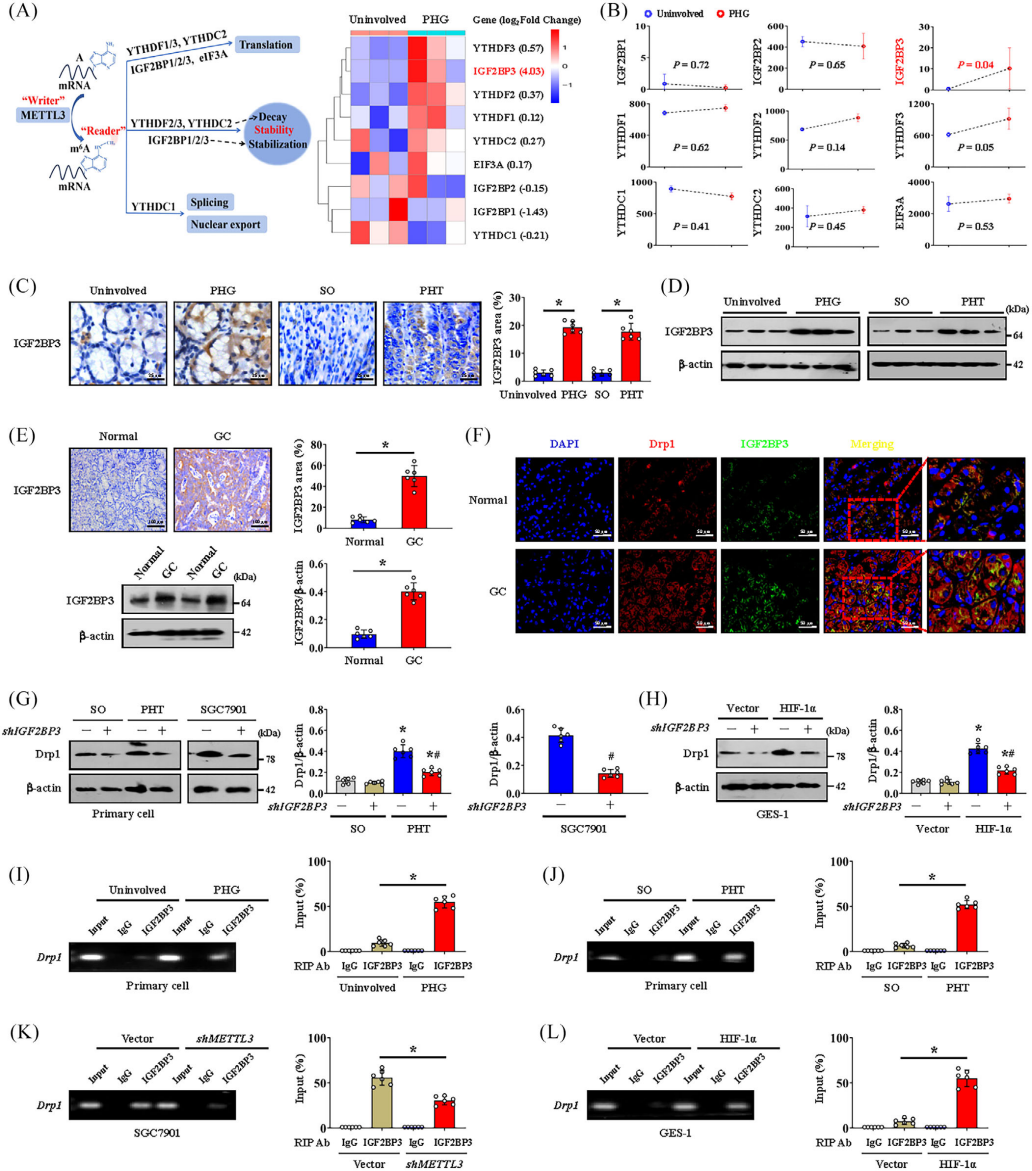
METTL3 regulates dynamin‐related protein 1 (Drp1) N6‐methyladenosine (m6A) modification and expression in an IGF2BP3‐dependent manner. (A) Schematic representation of the action of different ‘Readers’ (left panel). The heatmap showing the different ‘Readers’ expression levels in the uninvolved and portal hypertensive gastropathy (PHG) groups. (B) The expression levels of different Readers in the indicated groups were analysed via microarray analysis. *n* = 3 per group. (C) IGF2BP3 IHC staining (brown) in the related groups was presented. The area affected by IGF2BP3 was also presented. *n* = 6 per group. ^*^ *p* < .05. (D) The expression of IGF2BP3 was measured by western blotting. (E) The expression of IGF2BP3 in the normal and gastric cancer (GC) groups was detected by IHC staining (brown) and western blotting. The ratio of densitometry units of normalised IGF2BP3/β‐actin and the IGF2BP3 area were listed. *n* = 6 per group. ^*^ *p* < .05. (F) Double immunofluorescence (IF) staining of Drp1 (red) and IGF2BP3 (green) in the normal and GC groups was shown. Nuclei (blue) were counterstained with 4′,6‐diamidino‐2‐phenylindole dihydrochloride (DAPI). (G) Drp1 expression in the indicated groups after different treatments was measured by western blotting. The ratio of densitometry units of normalised Drp1/β‐actin was also analysed. *n* = 6 per group. ^*^ *p* < .05 versus sham operation (SO) mice, ^#^ *p* < .05 versus portal hypertension (PHT) mice or SGC7901 cells without *shIGF2BP3* transfection. (H) Drp1 expression in vector‐ and *HIF‐1α*‐transfected GES‐1 cells with or without *shIGF2BP3* transfection was measured by western blotting. The ratio of densitometry units of normalised Drp1/β‐actin was listed. *n* = 6 per group. ^*^ *p* < .05 versus SO mice, ^#^ *p* < .05 versus HIF‐1α‐transfected GES‐1 cells without *shIGF2BP3* transfection. (I) Agarose electrophoresis and RNA immunoprecipitation (RIP)‐qPCR using an anti‐IGF2BP3 antibody showing direct binding between the IGF2BP3 protein and Drp1 mRNA in primary epithelial cells isolated from PHG patients than that from healthy volunteers (uninvolved). ^*^ *p* < .05. (J) Agarose electrophoresis and RIP‐qPCR using an anti‐IGF2BP3 antibody revealing direct binding between the IGF2BP3 protein and Drp1 mRNA in primary epithelial cells isolated from the indicated mouse models. ^*^ *p* < .05. (K) Agarose electrophoresis and RIP‐qPCR using an anti‐IGF2BP3 antibody revealed that IGF2BP3 significantly bound to Drp1 mRNA in SGC7901 cells without *METTL3* knockdown (*shMETTL3* transfection). ^*^ *p* < .05. (L) Agarose electrophoresis and qPCR analysis of RIP assays using an anti‐IGF2BP3 antibody revealed the affinity of IGF2BP3 for Drp1 mRNA in vector‐ and *HIF‐1α*‐transfected GES‐1 cells. ^*^ *p* < .05. HIF‐1α, hypoxia‐inducible factor‐1α.

M6A methylation modifies downstream genes in a ‘Reader’‐dependent manner (Figure 5A). After searching our MeRIP‐seq data, we found that the expression of the ‘Reader’ gene IGF2BP3 exhibited the most significant difference and the highest upregulation ratio in PHT‐induced gastric mucosa compared to those in uninvolved healthy gastric mucosa (Figure 5A,B). Upregulated expression of IGF2BP3 was also confirmed in portal hypertensive gastric mucosa tissues and GC tissues from human and mouse samples (Figure 5C–E). Double IF staining revealed that Drp1 colocalised with IGF2BP3 in GC tissues, suggesting that m6A methylation of Drp1 might occur in an IGF2BP3‐dependent manner (Figure 5F). *IGF2BP3* knockdown by shRNA inhibited Drp1 expression in primary gastric mucosal epithelial cells isolated from the PHT group and in GC cells isolated from SGC7901 model mice (Figure 5G). HIF‐1α overexpression‐mediated Drp1 upregulation was also reversed by *IGF2BP3* knockdown (Figure 5H). We analysed tissues from human samples and mouse models, and agarose electrophoresis and RNA immunoprecipitation (RIP)‐qPCR assays demonstrated that IGF2BP3 could significantly bind to *Drp1* mRNA in gastric epithelial cells isolated from PHG patients and PHT mice (Figure 5I,J). Moreover, RIP‐qPCR analysis revealed that IGF2BP3 had a dramatically reduced affinity for *Drp1* mRNA in *METTL3*‐silenced (*shMETTL3*‐transfected) SGC7901 cells but not in SGC7901 cells without *shMETTL3* transfection (Figure 5K). Additionally, direct interaction between IGF2BP3 and *Drp1* mRNA was observed in HIF‐1α‐transfected GES‐1 cells but was hardly detected in vector‐transfected GES‐1 cells (Figure 5L). These findings indicated a crucial role for IGF2BP3 in facilitating HIF‐1α/METTL3‐mediated Drp1 m6A methylation. Generally, we concluded that HIF‐1α/METTL3‐mediated changes in Drp1 m6A methylation and expression are dependent on IGF2BP3.

### 3.6 HIF‐1α influences the metabolic profile and enhances glycolysis via LDHA

**FIGURE 6:**
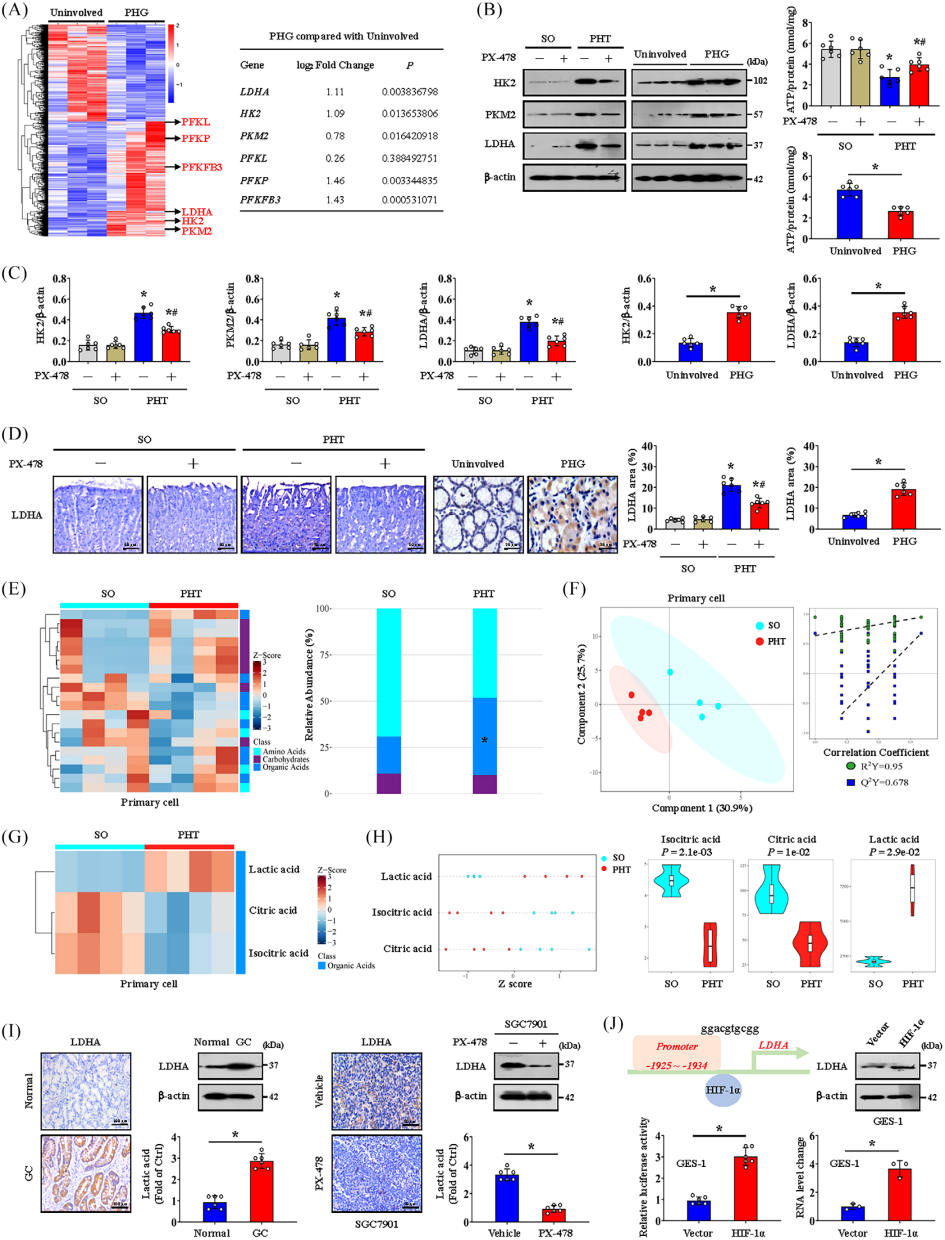
Hypoxia‐inducible factor‐1α (HIF‐1α) influences the metabolic profile and enhances glycolysis via lactate dehydrogenase A (LDHA). (A) Heatmap showing the differentially expressed genes associated with glycolysis in the uninvolved and portal hypertensive gastropathy (PHG) groups. *n* = 3 per group. (B) Hexokinase‐2 (HK2), pyruvate kinase M2 (PKM2) and LDHA levels in mouse models and clinical samples were measured by western blotting. The ATP concentrations were also analysed. *n* = 6 per group. ^*^ *p *< .05, ^#^ *p* < .05 versus portal hypertension (PHT) mice without PX‐478 treatment. (C) The ratio of densitometry units of normalised HK2/β‐actin, PKM2/β‐actin and LDHA/β‐actin was determined. *n* = 6 per group. ^*^ *p* < .05 versus sham operation (SO) mice or uninvolved groups, ^#^ *p* < .05 versus PHT mice without PX‐478 treatment. (D) LDHA IHC staining (brown) of mouse models and clinical sections was shown, and the area of LDHA was also analysed. *n* = 6 per group. ^*^ *p* < .05 versus SO mice or the uninvolved group, ^#^ *p* < .05 versus PHT mice without PX‐478 treatment. (E) Heatmap of an untargeted metabolomics strategy in primary gastric epithelial cells from the SO and PHT groups (left panel). *n* = 4 per group. The histogram revealed the differences in relative abundance between the SO and PHT groups (right panel). ^*^ *p* < .05. (F) A partial least squares discriminant analysis (PLS‐DA) model and correlation coefficient analysis showing the changes in metabolites in primary gastric epithelial cells. *n* = 4 per group. (G) Heatmap of metabolic alterations in primary epithelial cells showing significant differences in lactic acid, citric acid and isocitric acid between the SO group and PHT group. *n* = 4 per group. (H) The relative levels of lactic acid, isocitric acid and citric acid were presented and analysed via a *Z* score plot map and a Violin plot, respectively. *n* = 4 per group. (I) LDHA expression in the related groups was determined by IHC staining (brown) and western blotting analysis. *n* = 6 per group. ^*^ *p *< .05. (J) The binding sequence of HIF‐1α to the *LDHA* promoter. The LDHA protein levels in the vector‐ and *HIF‐1α*‐overexpressing groups were shown. The *LDHA* luciferase reporter activities and ChIP assays (the HIF‐1α‐associated complex was enriched in a specific region of the *LDHA* promoter) were also demonstrated. *n* = 3 per group. ^*^ *p *< .05. GC, gastric cancer.

Metabolic disorders are common in most diseases. Based on our RNA‐seq data and validation in gastric mucosa from patients with PHG and healthy volunteers, we found that multiple genes related to glycolysis, especially LDHA, PKM2 and HK2, were elevated in the PHG (Figure 6A,B). Furthermore, the levels of these key glycolytic enzymes were also elevated in PHT model mice but were reversed by PX‐478 (Figure 6B,C). The ATP levels in the gastric mucosa of PHT model and PHG were decreased, but this decrease could be reversed by PX‐478 in PHT model (Figure 6B). Elevated levels of LDHA, the most altered glycolytic enzyme, were measured in gastric mucosal tissues from clinical PHG patients and PHT model mice, and this increase could be repressed by PX‐478 in these mouse models (Figure 6D). In addition, metabolic analysis of primary gastric mucosal epithelial cells from SO or PHT mice was performed. Amino acid, carbohydrate and organic acid pathways were noted, and organic acid metabolism showed meaningful enrichment (Figure 6E,F). More precisely, lactic acid was apparently elevated in the gastric mucosa of PHT model, which was consistent with active glycolysis (Figure 6G,H). Additionally, elevated LDHA levels and lactic acid production were found in the GC and SGC7901 mouse models, and PX‐478 alleviated these changes in the SGC7901 mouse model (Figure 6I). Interestingly, dual‐luciferase and ChIP assays showed that HIF‐1α could also directly bind to the *LDHA* promoter and activate its expression (Figure 6J). Thus, HIF‐1α enhances glycolysis under hypoxia via LDHA in gastric mucosal diseases.

### 3.7 Blocking Drp1‐dependent mitochondrial fission represses glycolysis

**FIGURE 7:**
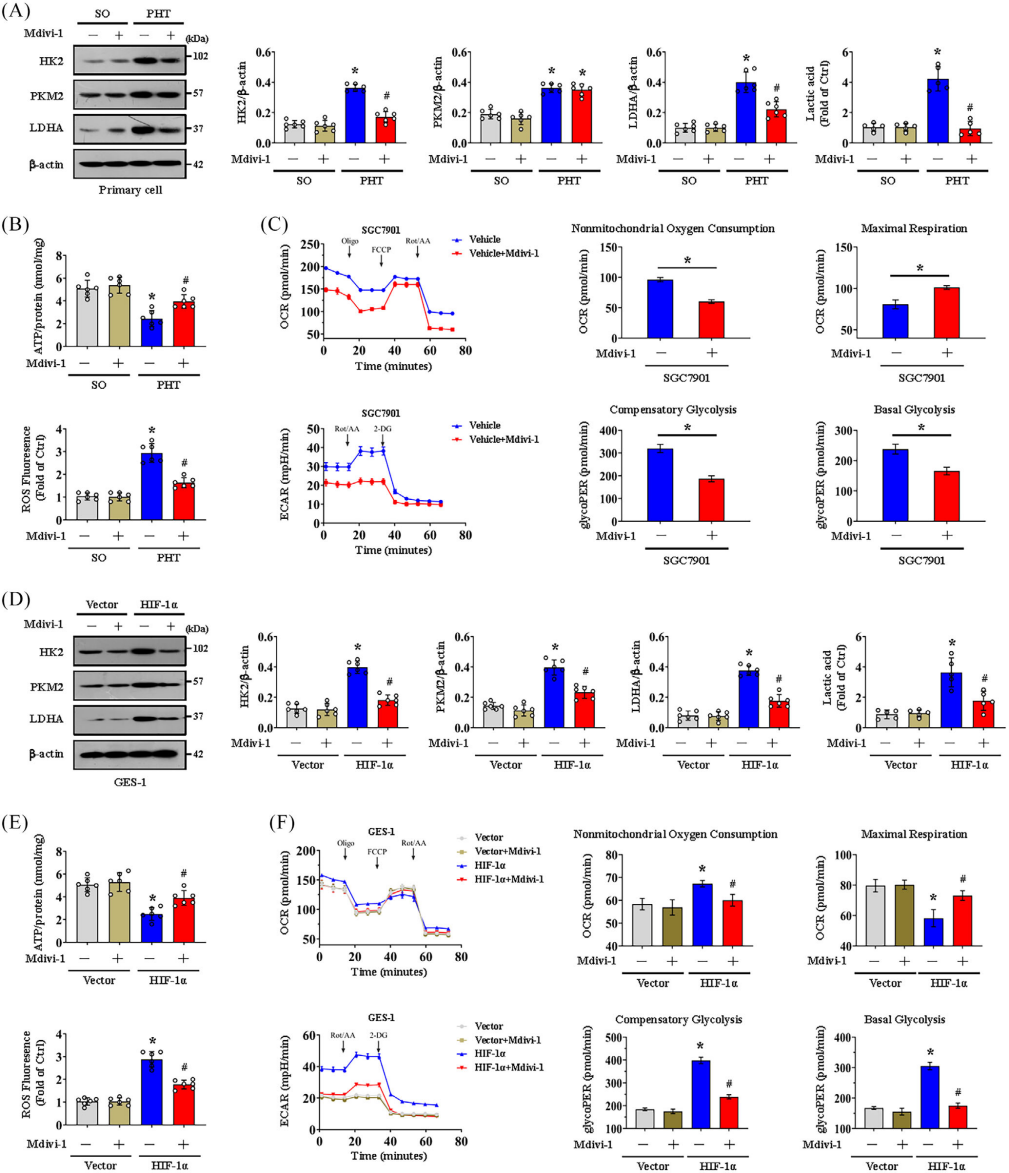
Blocking dynamin‐related protein 1 (Drp1)‐dependent mitochondrial fission represses glycolysis. (A) The hexokinase‐2 (HK2), pyruvate kinase M2 (PKM2) and lactate dehydrogenase A (LDHA) levels were measured by western blotting in primary gastric epithelial cells from the sham operation (SO) and portal hypertension (PHT) groups with or without a mitochondrial division inhibitor 1 (Mdivi‐1) treatment. The ratios of densitometric units of normalised HK2/β‐actin, PKM2/β‐actin and LDHA/β‐actin were determined via western blotting. The lactic acid concentration was also measured. *n* = 6 per group. ^*^ *p* < .05 versus SO mice, ^#^ *p* < .05 versus PHT mice without Mdivi‐1 treatment. (B) ATP concentrations and reactive oxygen species (ROS) levels were analysed in primary epithelial cells from the SO and PHT groups with or without Mdivi‐1 treatment. *n* = 6 per group. ^*^ *p *< .05 versus SO mice, ^#^ *p *< .05 versus PHT mice without Mdivi‐1 treatment. (C) Mitochondrial respiration (oxygen consumption rate, OCR) analysis of cells isolated from SGC7901 tumour model mice (with or without Mdivi‐1 treatment) was presented. *n* = 6 in each group. Glycolytic rate (extracellular acidification rate, ECAR) analysis of cells isolated from SGC7901 mouse tumour models (with or without Mdivi‐1 treatment) was also performed. ^*^ *p *< .05. (D) HK2, PKM2 and LDHA expression in vector‐ and *HIF‐1α*‐transfected GES‐1 cells with or without Mdivi‐1 treatment measured by western blotting. The ratios of densitometry units of normalised HK2/β‐actin, PKM2/β‐actin and LDHA/β‐actin were listed, and the lactic acid concentration was also measured. *n* = 6 per group. ^*^ *p* < .05 versus vector‐transfected GES‐1 cells, ^#^ *p* < .05 versus *HIF‐1α*‐transfected GES‐1 cells without Mdivi‐1 treatment. (E) ATP concentrations and ROS levels were determined in vector‐ and *HIF‐1α*‐transfected GES‐1 cells treated with or without Mdivi‐1. *n* = 6 per group. ^*^ *p* < .05 versus vector‐transfected GES‐1 cells, ^#^ *p* < .05 versus *HIF‐1α*‐transfected GES‐1 cells without Mdivi‐1 treatment. (F) OCR analysis of vector‐ and *HIF‐1α*‐transfected GES‐1 cells with or without Mdivi‐1 treatment was presented. ECAR of the indicated groups was also examined. ^*^ *p* < .05 versus vector‐transfected GES‐1 cells, ^#^ *p* < .05 versus *HIF‐1α*‐transfected GES‐1 cells without Mdivi‐1 treatment. FCCP, carbonyl cyanide‐4 (trifluoromethoxy) phenylhydrazone; HIF‐1α, hypoxia‐inducible factor‐1α; Oligo, oligomycin; Rot/AA, rotenone/antimycin A.

We also investigated whether Drp1‐dependent mitochondrial fission affects glycolytic process in gastric epithelial cells. The expression of the glycolytic proteins HK2, PKM2 and LDHA and the lactic acid concentration were obviously increased in the epithelial cells of PHT mice (Figure 7A), while HK2 and LDHA expression and the lactic acid concentration were decreased by Mdivi‐1 (Figure 7A). The ATP production was improved and ROS levels were decreased by Mdivi‐1 in primary epithelial cells from PHT models (Figure 7B). We found that nonmitochondrial oxygen consumption and maximum respiration were reversed by Mdivi‐1 in cells isolated from SGC7901 mouse gastric graft tumour models by detecting the OCR value (Figure 7C). The glycolytic rate determined by the ECAR level was also analysed, and compensatory and basal glycolysis were found to be decreased in cells isolated from SGC7901 tumour model mice treated with Mdivi‐1 (Figure 7C). The HK2, PKM2 and LDHA levels and the lactic acid concentration were also increased in *HIF‐1α*‐transfected GES‐1 cells but were obviously decreased by Mdivi‐1 (Figure 7D). Mdivi‐1 also enhanced ATP production and inhibited ROS generation in *HIF‐1α*‐transfected GES‐1 cells (Figure 7E). We verified that increased nonmitochondrial oxygen consumption and reduced maximum respiration occurred in HIF‐1α‐transfected GES‐1 cells by detecting the OCR, and these changes could be reversed by Mdivi‐1 (Figure 7F). The ECAR assay further revealed increased compensatory and basal glycolysis in *HIF‐1α*‐transfected GES‐1 cells, which were blocked by Mdivi‐1 (Figure 7F). These data indicate that Drp1‐dependent mitochondrial fission enhances glycolysis, while blockade of mitochondrial fission can attenuate the glycolytic process.

### 3.8 Drp1‐dependent mitochondrial fission associated with dysfunction of the mitochondrial electron transport chain contributes to oxidative stress in both PHG and GC

**FIGURE 8:**
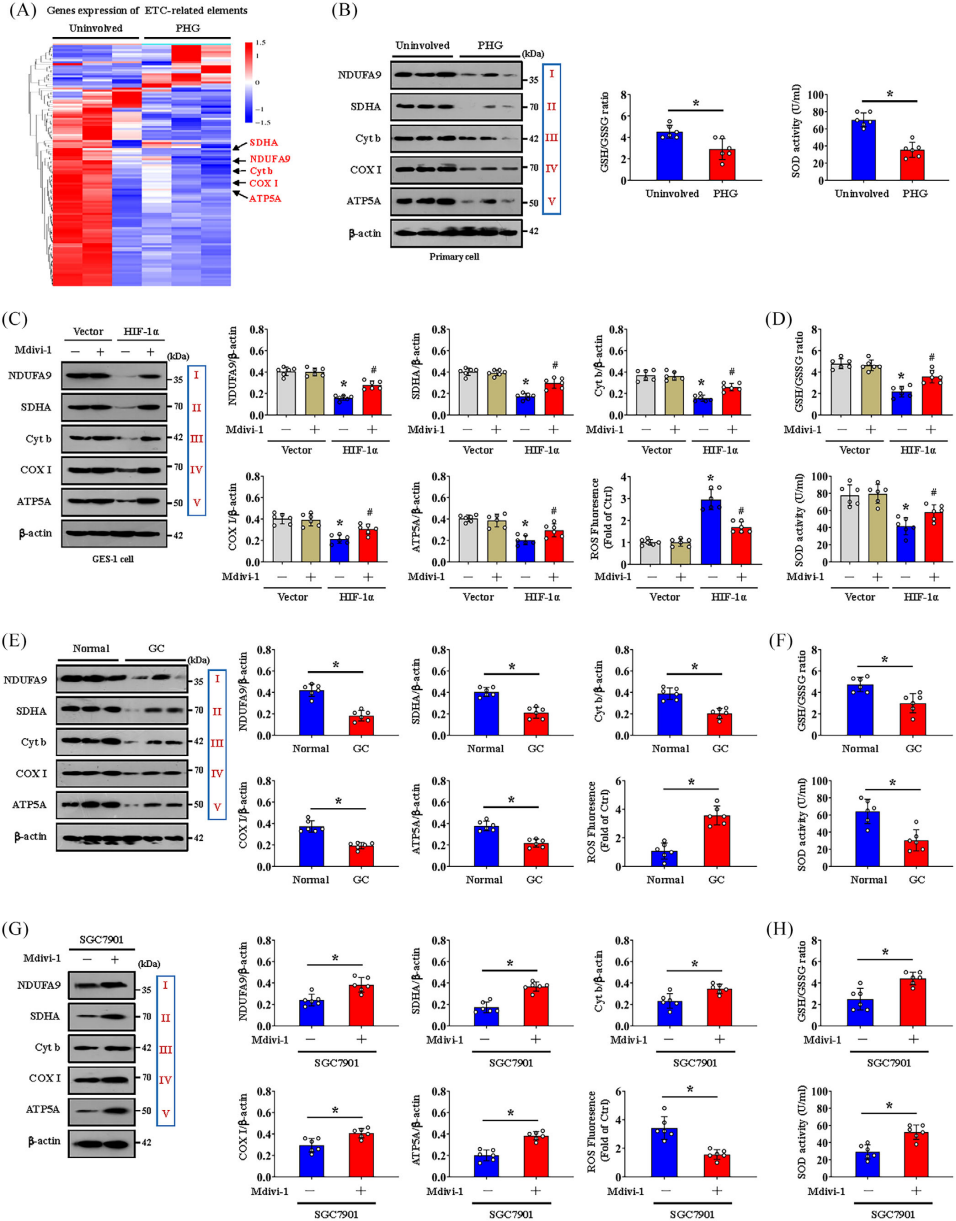
Dynamin‐related protein 1 (Drp1)‐dependent mitochondrial fission associated with dysfunction of the mitochondrial electron transport chain contributes to oxidative stress in both portal hypertensive gastropathy (PHG) and gastric cancer (GC). (A) Two‐dimensional hierarchical clustering results of the genes of electron transport chain (ETC)‐related elements between PHG patients (as PHG) and healthy volunteers (as uninvolved). *n* = 3 per group. (B) Western blotting analysis (left panel) of protein levels of representative ETC complex subunits (NADH ubiquinone oxidoreductase subunit A9 [NDUFA9], succinate dehydrogenase complex flavoprotein subunit A [SDHA], cytochrome b [Cyt b], cyclooxygenase I [COX I] and alpha subunit of ATP synthase [ATP5A]) in the primary epithelial cells of PHG patients and healthy volunteers (uninvolved). The activity of the antioxidant enzyme superoxide dismutase (SOD) and the ratio of reduced (glutathione, GSH) to oxidised (glutathione disulphide, GSSG) states (GSH/GSSG) were detected by assay kits (right panel). *n* = 6 per group. ^*^ *p* < .05. (C) Western blotting analysis of protein levels of representative ETC complex subunits (NDUFA9, SDHA, Cyt b, COX I and ATP5A) in vector‐ and *HIF‐1α*‐transfected GES‐1 cells with or without mitochondrial division inhibitor 1 (Mdivi‐1) treatment. The ratios of the normalised NDUFA9/β‐actin, SDHA/β‐actin, Cyt b/β‐actin, COX I/β‐actin and ATP5A/β‐actin densitometric units and the levels of reactive oxygen species (ROS) were also analysed. *n* = 6 per group. ^*^ *p* < .05 versus vector‐transfected GES‐1 cells, ^#^ *p* < .05 versus *HIF‐1α*‐transfected GES‐1 cells without Mdivi‐1 treatment. (D) SOD activity and the GSH/GSSG ratio in the indicated groups were detected by assay kits. *n* = 6 per group. ^*^ *p* < .05 versus vector‐transfected GES‐1 cells, ^#^ *p* < .05 versus *HIF‐1α*‐transfected GES‐1 cells without Mdivi‐1 treatment. (E) Western blot showing the protein levels of representative ETC complex subunits (NDUFA9, SDHA, Cyt b, COX I and ATP5A) in the normal and GC groups. The ratios of the normalised NDUFA9/β‐actin, SDHA/β‐actin, Cyt b/β‐actin, COX I/β‐actin and ATP5A/β‐actin densitometric units and the levels of ROS were also examined. *n* = 6 per group. ^*^ *p* < .05. (F) SOD activity and the GSH/GSSG ratio of the normal and GC groups were analysed. *n* = 6 per group. ^*^ *p* < .05. (G) Western blotting detection of representative ETC complex subunit protein levels in cells isolated from SGC7901 xenograft model mice. The ratios of the normalised NDUFA9/β‐actin, SDHA/β‐actin, Cyt b/β‐actin, COX I/β‐actin and ATP5A/β‐actin densitometric units and the levels of ROS were also examined. *n* = 6 per group. ^*^ *p* < .05. (H) SOD activity and the GSH/GSSG ratio in cells isolated from SGC7901 xenograft model mice were analysed. *n* = 6 per group. ^*^ *p* < .05. HIF‐1α, hypoxia‐inducible factor‐1α; NADH, nicotinamide adenine dinucleotide.

Mitochondrial ATP generation and ROS production are intimately related to the function of the ETC. To explore whether a defect in the ETC affects mtROS production under conditions of Drp1‐dependent mitochondrial fission and altered glycolysis status, we examined the transcriptomes of gastric mucosal tissues to assess the ETC profiles and found that multiple ETC‐related elements were modulated between PHG patients and uninvolved volunteers (Figure 8A and Table S1). Western blotting analysis of representative ETC complex subunits revealed that the levels of NDUFA9, SDHA, Cyt b, COX I and ATP5A were decreased in both PHG patients and GC patients (Figure 8B,E), and the generation of ROS was increased in GC patients (Figure 8E). The activity of the antioxidant enzyme superoxide dismutase (SOD) and the ratio of reduced (GSH, glutathione) to oxidised (GSSG, glutathione disulphide) states (GSH/GSSG) were also decreased in both PHG patients and GC patients (Figure 8B,F). Moreover, the protein levels of NDUFA9, SDHA, Cyt b, COX I and ATP5A were markedly repressed in both *HIF‐1α*‐transfected GES‐1 cells and cells isolated from SGC7901 xenograft mouse models (Figure 8C,G). Mdivi‐1 not only reversed the defects in the expression of the abovementioned ETC complex subunits (Figure 8C,G), but also decreased ROS levels and enhanced SOD activity and the GSH/GSSG ratio in both *HIF‐1α*‐transfected GES‐1 cells and cells isolated from SGC7901 xenograft mouse models (Figure 8C,D,G,H). In summary, Drp1‐dependent mitochondrial fission associated with alterations in antioxidant enzyme activity and dysfunction of the ETC contributes to oxidative stress in both the PHG and GC.

### 3.9 Mitochondrial dysfunction‐induced oxidative stress activates the NLRP3 inflammasome to contribute to the development of gastric mucosal diseases

**FIGURE 9:**
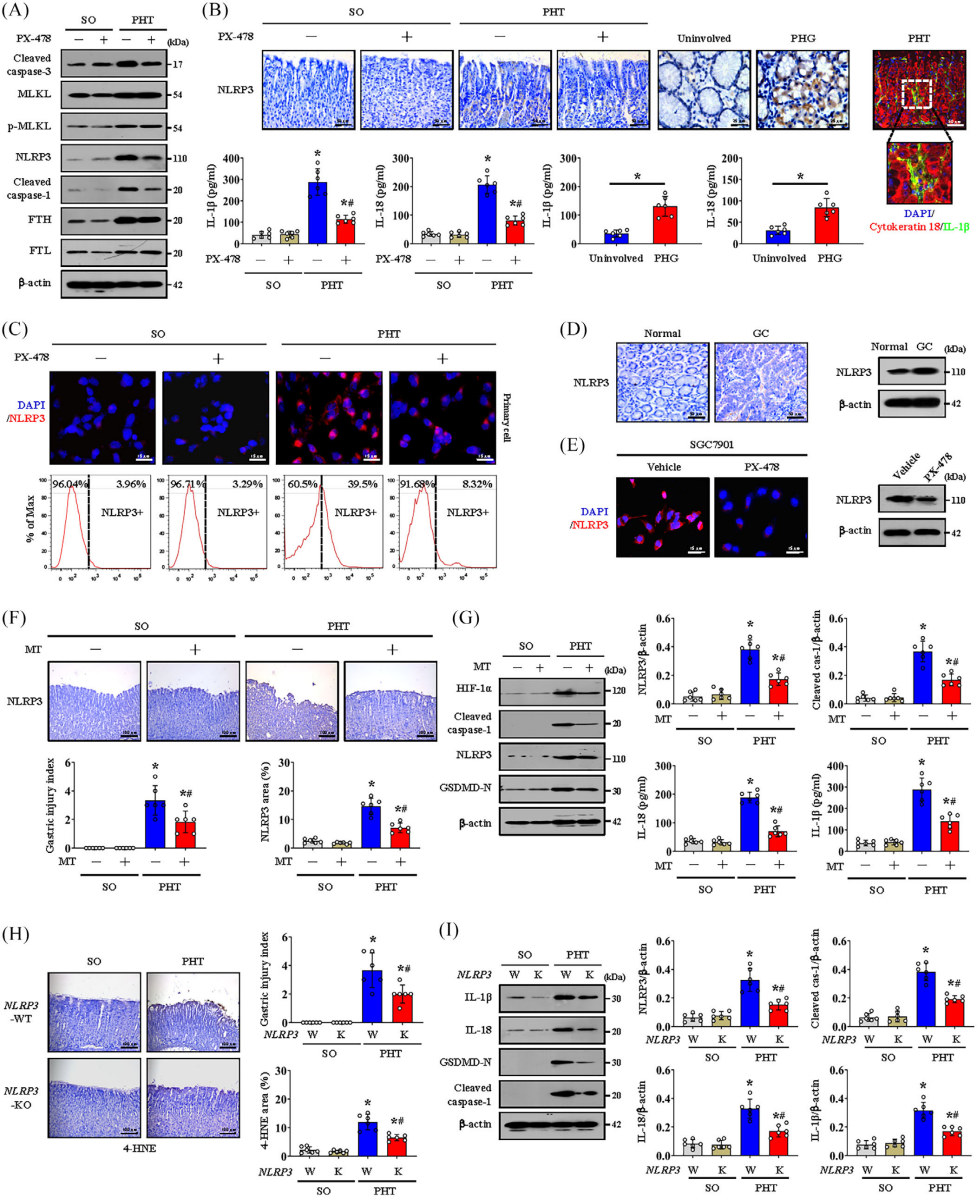
Mitochondrial dysfunction‐induced oxidative stress activates the NLRP3 inflammasome to contribute to the development of gastric mucosal diseases. (A) The expression of cleaved caspase‐3, MLKL, p‐MLKL, NLRP3, cleaved caspase‐1, FTH and FTL in the sham operation (SO) and portal hypertension (PHT) groups with or without PX‐478 treatment. (B) IHC staining of NLRP3 (brown) in the indicated groups. Histograms showing the levels of interleukin (IL)‐1β and IL‐18 in the indicated groups determined by enzyme‐linked immunosorbent assay (ELISA). *n* = 6 per group. ^*^ *p* < .05 versus SO mice or uninvolved groups, ^#^ *p* < .05 versus PHT mice without PX‐478. Co‐staining for cytokeratin 18 (red) and IL‐1β (green) was also shown, and nuclei (blue) were counterstained with 4′,6‐diamidino‐2‐phenylindole dihydrochloride (DAPI). (C) Immunofluorescence (IF) staining of NLRP3 (red) in primary cells isolated from the SO and PHT groups. Flow cytometric analysis was used to detect NLRP3‐positive (NLRP3+) and NLRP3‐negative (NLRP3‒) gastric epithelial cells from the above groups. (D) NLRP3 IHC staining (brown) and protein detection in normal and gastric cancer (GC) gastric tissues. (E) NLRP3 IF staining (red) and protein levels in cells isolated from SGC7901 xenografts of nude mice with or without PX‐478 treatment were revealed. (F) NLRP3 staining (brown) and the gastric injury index of model mice treated with or without mito‐TEMPO (MT). *n* = 6 per group, ^*^ *p* < .05 versus the SO group, ^#^ *p* < .05 versus the PHT group without MT treatment. (G) The expression of hypoxia‐inducible factor‐1α (HIF‐1α), cleaved caspase‐1, NLRP3 and N‐terminal cleaved gasdermin D (GSDMD‐N) was analysed by western blotting (left panel). The ratio of densitometry units of normalised NLRP3 and cleaved caspase‐1 to β‐actin was determined (upper panel). The concentrations of IL‐1β and IL‐18 determined by ELISA were also shown (lower panel). *n* = 6 per group. ^*^ *p *< .05 versus the SO group, ^#^ *p* < .05 versus the PHT group without MT treatment. (H) Expression of 4‐hydroxynonenal (4‐HNE) in *NLRP3*‐WT (W) or *NLRP3*‐KO (K) mice according to IHC staining (brown). The gastric injury index and 4‐HNE area (%) were also analysed. *n* = 6 per group. ^*^ *p* < .05 versus SO mice, ^#^ *p *< .05 versus PHT *NLRP3*‐WT mice. (I) The expression of IL‐1β, IL‐18, cleaved caspase‐1 and GSDMD‐N was measured by western blotting. The densitometry units of the corresponding proteins were analysed. *n* = 6 per group. ^*^ *p* < .05 versus SO mice, ^#^ *p *< .05 versus PHT *NLRP3*‐WT mice. PHG, portal hypertensive gastropathy.

To further analyse the cell death outcomes of hypoxia‐induced mitochondrial dysfunction, we evaluated common markers of apoptosis, necrotic apoptosis, ferroptosis and pyroptosis in gastric mucosal lesions in PHT and GC. We found that the NLRP3 and cleaved caspase‐1 levels, but not those of MLKL, FTH or FTL, were increased in both *HIF‐1α*‐transfected GES‐1 cells and cells isolated from SGC7901 xenograft model mice and decreased following PX‐478 treatment (Figure S5A,B). Similarly, the cleaved caspase‐3 level and p‐MLKL level were decreased following PX‐478 treatment in the cells from SGC7901 xenograft mice, and the p‐MLKL level was enhanced in *HIF‐1α*‐transfected GES‐1 cells (Figure S5A,B), revealing that in addition to NLRP3‐mediated pyroptosis, other types of cell death, such as apoptosis or necrotic apoptosis, may also play a partial role in HIF‐1α‐regulated cell death. Moreover, the upregulation of NLRP3 and cleaved caspase‐1 in primary cells isolated from PHG patients and GC patients was obviously repressed by PX‐478 treatment (Figure S5C,D). Although the expression of cleaved caspase‐3, p‐MLKL and FTH was also found to be modulated in primary cells isolated from PHG and GC tissues, PX‐478 treatment did not influence their status (Figure S5C,D). These data suggest that NLRP3 inflammasome‐mediated pyroptosis, rather than other types of cell death, mainly contributes to the development of gastric mucosal lesions modulated by HIF‐1α signalling. Moreover, the activation of the inflammasome NLRP3, which was associated with the secretion of the inflammatory mediators IL‐18 and IL‐1β, was significantly enhanced in PHT‐mediated gastric mucosal injury in both humans and mice, and PX‐478 repressed this process (Figure 9A,B). Coimmunostaining of IL‐1β and CK18 revealed that damaged gastric mucosal epithelial cells were surrounded by these inflammatory factors (Figure 9B). The expression of NLRP3 was further confirmed in primary gastric epithelial cells isolated from the SO or PHT groups that received PX‐478 treatment and those that did not receive PX‐478 treatment, and fluorescence labelling and flow cytometry analysis revealed that PX‐478 decreased the proportion of NLRP3‐positive cells in the PHT group (Figure 9C). Moreover, both NLRP3 expression and IL‐18 and IL‐1β levels were increased in the GC and SGC7901 mouse models (Figures 9D,E and S6A) but decreased by PX‐478 treatment in the SGC7901 mouse model (Figures 9E and S6B). We subsequently found that the ROS scavenger MT inhibited NLRP3 inflammasome activation and alleviated the gastric injury index in PHT models (Figure 9F,G). MT also inhibited cleaved caspase‐1 and N‐terminal cleaved gasdermin D (GSDMD‐N) expression and reduced IL‐18 and IL‐1β secretion (Figure 9G). Suppression of ROS by MT reduced tumour size (average tumour size: 14.38 mm in SGC7901 xenograft mouse models without MT vs. 8.90 mm in those with MT) and tumour weight (5.27% in SGC7901 xenograft mouse models without MT vs. 2.42% in those with MT) (Figure S6C). MT also repressed GC cell proliferation (Ki67 index: 34.98% in SGC7901 xenograft mouse models without MT vs. 12.02% in those with MT) and attenuated NLRP3 activation (NLRP3 area: 18.32% in SGC7901 xenograft mouse models without MT vs. 11.07% in those with MT) in SGC7901 mouse tumour models (Figure S6C).

The PHT models established by adopting *NLRP3*‐WT and *NLRP3*‐KO mice were used. *NLRP3*‐KO alleviated gastric mucosal injury and 4‐HNE levels and inhibited the cleaved caspase‐1/GSDMD‐N pathway to reduce the release of IL‐18 and IL‐1β (Figure 9H,I). Similarly, the NLRP3 inhibitor MCC950 decreased tumour size (average tumour size: 14.77 mm in SGC7901 xenograft mouse models without MCC950 vs. 8.47 mm in those with MCC950) and tumour weight (5.40% in SGC7901 xenograft mouse models without MCC950 vs. 2.82% in those with MCC950) in SGC7901 mouse tumour models (Figure S6D,E). MCC950 also repressed GC cell proliferation (Ki67 index: 34.60% in SGC7901 xenograft mouse models without MCC950 vs. 11.57% in those with MCC950) and NLRP3 activation (NLRP3 area: 17.47% in SGC7901 xenograft mouse models without MCC950 vs. 10.87% in those with MCC950) in SGC7901 mouse tumour models (Figure S6D,E).

**FIGURE 10:**
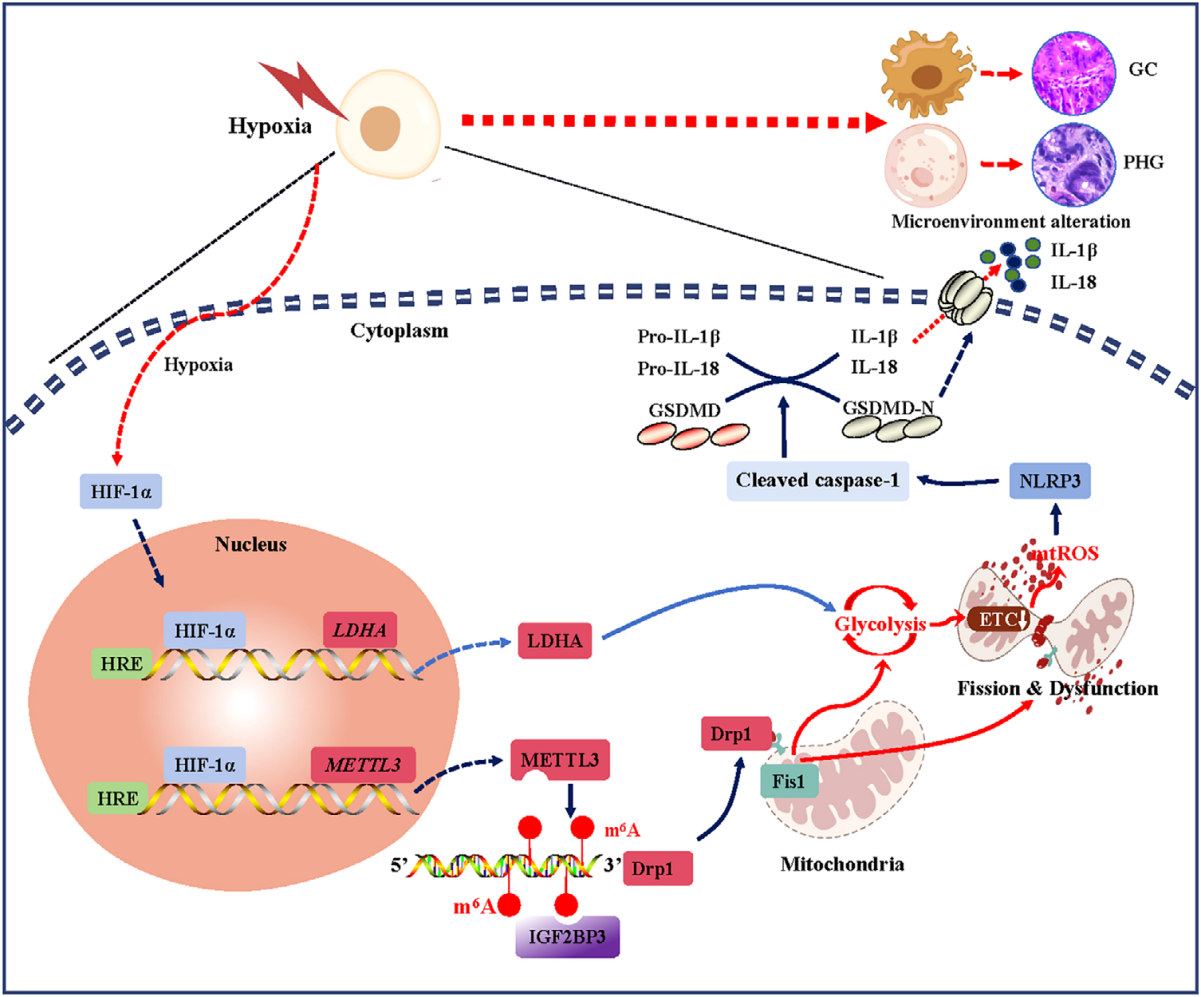
Schematic diagram of hypoxia‐inducible factor‐1α (HIF‐1α)‐regulated mitochondrial fission and dysfunction in the development of gastric mucosal diseases under hypoxia.

In conclusion, HIF‐1α enhances mitochondrial dysfunction through Drp1‐dependent mitochondrial fission and influences the metabolic profile by enhancing glycolysis to increase mtROS production, which can trigger NLRP3 inflammasome activation and mucosal microenvironment alterations to contribute to the development of benign and malignant gastric mucosal lesions (Figure 10).

## 4 DISCUSSION

Hypoxia is an important characteristic of gastric mucosal diseases, ranging from mucosal inflammatory reactions to GC.^29^, ^30^ PHT is indicated by a portal pressure gradient greater than 5 mmHg, namely, the difference in pressure between the portal vein and the hepatic veins, which mainly results from liver cirrhosis,^31^ and PHT‐related gastric mucosal lesions are commonly defined as complications in patients with PHT.^3^ And PHG and GC are the most representative benign and malignant gastric mucosal diseases characterised by hypoxia.^32^, ^33^ To identify the hypoxic situation of the gastric mucosa caused by PHT and GC, gastric tissues from patients clinically diagnosed with PHG and GC were collected, PHT‐related gastric mucosal injury models and SGC7901 gastric graft tumour models were constructed, and the hypoxic state in the gastric mucosa was confirmed in PHT and GC. Moreover, HIF‐1α was significantly increased and concentrated in the nucleus to exert its function as a transcription factor and was positively correlated with the degree of gastric mucosal injury. Because mitochondria are strongly regulated by hypoxia, they sense O_2_ concentrations and initiate cellular responses to hypoxia.^34^ In response to hypoxia and the upregulation of HIF‐1α, mitochondria exhibit morphological abnormalities and functional impairment, leading to enhanced oxidative stress and ROS release in GC cells and injured gastric mucosal epithelial cells induced by PHT.

Stable mitochondrial fission/fusion is critically involved in mitochondrial quality control, whereas excessive mitochondrial fission may impair mitochondrial functions. Drp1 is a major component of mitochondrial fission and acts as a marker of mitochondrial dynamics.^35^ Four outer mitochondrial membrane adaptors anchor Drp1, Mff, Fis1 and mitochondrial division factors 49 and 51 (Mid49 and Mid51), and this process is primarily driven by Drp1 and Fis1.^36^, ^37^ In our study, Drp1 and Fis1 expression was elevated in injured gastric mucosa under hypoxia, and Drp1 interacted with Fis1 in mitochondria to activate mitochondrial fission and oxidative stress. PX‐478‐mediated HIF‐1α inhibition and Mdivi‐1‐mediated Drp1 inhibition both restored mitochondrial function to a certain extent, which alleviated PHT‐induced gastric mucosal injury and blocked GC cell proliferation, suggesting that HIF‐1α mediated mitochondrial dysfunction via Drp1‐dependent mitochondrial fission. Based on the important role of protein oligomerisation in mitochondrial fission, we also analysed the oligomeric states of Drp1, Fis1, Mff, Mid49, Mid51, Mfn1, Mfn2 and OPA1 and found that the oligomerisation of Drp1 and Fis1 was enhanced in gastric mucosal lesions in PHT and SGC7901 xenograft mouse models. The oligomerisation of Mff, Mid49 and Mid51 rather than that of Mfn1, Mfn2 and OPA1 in the gastric epithelial cells of PHT patients was slightly increased, and the oligomeric states of Mff, Mid49, Mid51 and Mfn1 were not modulated by PX‐478 treatment in SGC7901 xenograft mouse models. Drp1 oligomerisation always enhances mitochondrial fission,^26^ and Fis1 oligomers drive Drp1 oligomerisation via promoting Mid51 oligomerisation.^27^ Our findings revealed that enhanced Drp1 and Fis1 oligomerisation participates in HIF‐1α‐regulated mitochondrial fission in both PHT and GC.

As an epitranscriptomic modification, the m6A modification of RNA is widely involved in the regulation of cell renewal and differentiation and metabolism during the progression of various inflammatory and cancerous diseases.^38^ However, the specific mechanism by which m6A modification mediates mitochondrial dysfunction under hypoxic gastric condition is still unknown. Therefore, MeRIP‐seq analysis of normal and PHT‐induced gastric mucosa tissues revealed that m6A modification plays an important role in gastric mucosal lesions in PHT. More critically, the m6A level of Drp1 was increased. Furthermore, we found that the m6A modification of Drp1 was dependent on the ‘Writer’ METTL3 and ‘Reader’ IGF2BP3 and was strictly regulated by HIF‐1α under hypoxia. The same results were also demonstrated in GC cells and gastric mucosal tissues with cancerous lesions. Similar to our findings, a previous study showed that METTL3 promoted GC development wherein METTL3 catalysed the m6A methylation of growth arrest specific 5 (GAS5) in a YTHDF2‐mediated manner to enhance Drp1‐mediated mitochondrial fission.^39^, ^40^ Furthermore, PX‐478 can alleviate the m6A modification mediated by the HIF‐1α/METTL3/IGF2BP3 pathway to inhibit the interaction between Drp1 and Fis1 and mitochondrial fission/dysfunction. In addition, *METTL3* mutation or *IGF2BP3* knocking down inhibited m6A modification and decreased Drp1 protein levels to rescue mitochondrial function in PHT and GC nude mouse models.

Glycolysis is an important metabolic pathway controlled by many glycolytic enzymes, and previous reports have confirmed that gastric mucosal diseases utilise glycolysis to meet energy demands.^41^, ^42^, ^43^ Based on a transcriptional sequencing assay of clinical PHT‐induced injured gastric mucosal tissues combined with energy metabolism analysis of primary gastric mucosal epithelial cells from PHT model mice, we found that glycolysis was activated and regulated by the key rate‐limiting enzyme LDHA in the gastric mucosa of PHT models under hypoxia. Elevated glycolysis and LDHA were also measured in GC cells and gastric mucosal tissues with cancerous lesions under hypoxic condition. According to a recent report, stress signals (including glycolysis, inflammatory factors and hypoxia) can increase mitochondrial fission,^44^ and latent membrane protein 1‐mediated mitochondrial fission can also drive a metabolic shift to glycolysis.^35^ In the HIF‐1α‐dominated hypoxic state, LDHA‐mediated glycolytic activation and METTL3/IGF2BP3‐dependent increase in Drp1 m6A methylation jointly aggravated mitochondrial fission, dysfunction and ROS production. Mechanistically, HIF‐1α dimerises with HIF‐1β and binds to the hypoxia response element under hypoxia to control gene expression.^45^ Dual luciferase reporter plasmids and chromatin immunoprecipitation assays indicated that HIF‐1α can directly bind to the *LDHA* and *METTL3* promoters and increase their expression.

Energy deficiency diverts NLRP3‐dependent pyroptosis to NLRP3‐independent necrosis upon the addition of a K^+^ efflux inducer (extracellular ATP and the bacterial toxin nigericin), which is dampened by high‐energy intermediates such as fructose 1,6‐bisphosphate. Notably, cell death induced by K^+^ efflux and energy metabolism blockade is distinct from pyroptosis, apoptosis, necroptosis or ferroptosis.^46^ Based on our energy metabolism analysis, we showed that HIF‐1α not only mediated Drp1‐dependent mitochondrial fission and mitochondrial dysfunction in gastric mucosal diseases but also influenced energy metabolism and metabolic profiles by altering glycolysis under hypoxic condition and that blockade of Drp1‐dependent mitochondrial fission attenuated the glycolytic process. These data revealed that, in addition to HIF‐1α‐modulated glycolysis by LDHA, Drp1‐dependent mitochondrial fission/dysfunction also enhanced glycolysis, and blockade of mitochondrial fission attenuated the glycolytic process. Mitochondrial ATP generation and ROS production are intimately related to the function of the ETC, and nicotinamide adenine dinucleotide and flavin adenine dinucleotide, which are supplied by glycolysis, translocate to the ETC, each of which can donate a pair of electrons to the ETC.^47^ ETC dysfunction leads to the premature leakage of electrons from complexes to enhance mtROS generation, during this process, antioxidant systems are important for the regulation of mtROS and redox species.^47^ Based on these findings, we also found that Drp1‐dependent mitochondrial fission and enhanced glycolysis were associated with alterations in antioxidant enzyme activity and dysfunction of the ETC, which ultimately resulted in massive mtROS production. Increased mtROS can further promote mtDNA damage, the oxidation of mitochondrial proteins and mitochondrial dysfunction, in turn causing the massive production of mtROS.^48^ ox‐mtDNA was quantified by the 8‐OHdG content of mtDNA, and we revealed that mtROS induced mtDNA damage in gastric mucosal diseases. These data suggest that Drp1‐dependent mitochondrial fission/dysfunction caused by HIF‐1α alters energy metabolism and enhances glycolysis, thus producing massive amounts of mtROS through defects in the ETC and antioxidant systems in both PHG and GC.

Energy metabolism deficiency can also affect K^+^ channels and other kinases phosphorylation to regulate ROS generation.^46^ Some reports highlight the multifaceted interaction of HIF‐1α with cell death processes, including apoptosis, necrosis, autophagy, ferroptosis and pyroptosis.^49^ In our study, we further analysed the cell death outcomes of mitochondrial dysfunction‐mediated ROS production by HIF‐1α under hypoxia by evaluating the possibility of apoptosis, necrotic apoptosis, ferroptosis and pyroptosis in gastric mucosal lesions in PHT and GC, and we revealed that the protein levels of NLRP3 and cleaved caspase‐1, rather than those of MLKL, FTH and FTL, were increased in both *HIF‐1α*‐transfected GES‐1 cells and cells isolated from SGC7901 xenograft mouse models but could be decreased by PX‐478 treatment in cells from SGC7901 xenograft mice. Moreover, the upregulated NLRP3 and cleaved caspase‐1 levels in primary cells isolated from PHG patients and GC patients were decreased by PX‐478 treatment. Although the levels of cleaved caspase‐3, p‐MLKL, FTH and FTL were also found to be regulated in PHG and GC tissues, PX‐478 did not influence their status. These data highlighted the critical role of NLRP3 inflammasome‐mediated pyroptosis in cell death and gastric mucosal lesions under HIF‐1α‐regulated mitochondrial dysfunction and energy metabolism alteration (enhanced glycolysis). Consistent with our current results, HIF‐1α has been implicated in the regulation of inflammatory responses and cell death pathways, particularly NLRP3 inflammasome‐associated pyroptosis.^49^, ^50^, ^51^ HIF‐1α appears to regulate the expression of NLRP3, and mitophagy can suppress pyroptosis by blocking the HIF‐1α/NLRP3 pathway under hypoxia.^50^ miRNA‐18a inhibits HIF1‐α/NLRP3 signalling to hinder pyroptosis and impair osteogenic differentiation.^51^ HIF‐1α‐mediated glycolysis deficiency also alleviates NLRP3‐related inflammation, pyroptosis and oxidative stress.^52^ These reports revealed that HIF‐1α interacts with specific proteins, signalling pathways and genetic regulators to promote cell death processes, especially NLRP3 signalling‐dependent pyroptosis. Whether and how other forms of cell death, such as apoptosis, necrotic apoptosis and ferroptosis, are induced by energy metabolism alterations and mitochondrial dysfunction‐mediated ROS production under hypoxia in PHG and GC tissues warrants further investigation.

Inflammation affects all stages of tumourigenesis, and the activation of the NLRP3 inflammasome is the key to causing acute and chronic inflammation.^53^ Inflammasome activation requires priming by proinflammatory signals or oxidative stress products, and ROS plays a key role in NLRP3 inflammasome activation and inflammasome complex formation.^54^ Some studies have shown that the oxidation of mitochondrial DNA by mtROS is an inducer of NLRP3 inflammasome activation.^46^ The mitochondria within a cell are a major source of endogenous ROS and can be important triggers for inflammation and tissue injury, as ROS production activates the NLRP3 inflammasome and IL‐1β secretion through increased levels of thioredoxin‐interacting protein.^55^ In addition, the NLRP3 inflammasome senses mitochondrial dysfunction and is positively regulated by ROS derived from dysfunctional mitochondria.^56^ Additionally, we verified that the ROS scavenger MT inhibited NLRP3 inflammasome activation and alleviated the gastric injury index in PHT models, and MT also repressed GC proliferation and attenuated NLRP3 activation in SGC7901 mouse tumour models. However, the NLRP3 inflammasome is unlikely to be activated by ROS directly. NLRP3 can be activated by extracellular stimuli that induce K^+^ efflux, such as ATP released from dying cells.^46^, ^57^ According to our results, aberrant ATP levels in the gastric mucosa of PHT models and PHG patients under hypoxic condition may be involved in NLRP3 inflammasome activation, gastric mucosal inflammation and lesion formation, and these effects could be reversed by HIF‐1α inhibition with PX‐478. A decrease in mitochondrial membrane potential and increased intracellular Ca^2+^ can also be NLRP3 activators that elicit a particular form of mitochondrial damage.^56^ In this regard, we showed that *HIF‐1α* overexpression also decreased the membrane potential, disturbed mitochondrial Ca^2+^ homeostasis and enhanced oxidative stress in gastric epithelial cells. These changes may cause the release of fragmented mtDNA, which is then converted to an oxidised form (ox‐mtDNA) and serves as the ultimate NLRP3 ligand for its activation.^58^ On the other hand, PKM2 upregulation or its translocation to the nucleus may be required for NLRP3 inflammasome activation.^59^ Based on our RNA‐seq and local data from the gastric mucosa of PHG patients and healthy volunteers, we found that PKM2 was elevated in PHG under hypoxia, and the protein levels of multiple elements related to glycolysis, including LDHA, PKM2 and HK2, were elevated in PHG, which could be reversed by PX‐478 or Mdivi‐1. Active PKM2 was reported to activate NLRP3, and dimeric PKM2 bound with HIF‐1α promoted pro‐IL‐1β transcription and facilitated NLRP3 and apoptosis‐associated speck‐like CARD‐domain protein (ASC) assembly under hypoxic condition.^60^ These findings suggest that multiple factors in different pathophysiological situations contribute to NLRP3 inflammasome activation by modulating various signals or pathways. In our study, ROS generated from mitochondrial dysfunction and altered energy metabolism (enhanced glycolysis) by HIF‐1α under hypoxia ultimately triggered NLRP3 inflammasome activation and mucosal microenvironment alterations, thus leading to epithelial pyroptosis and the development of gastric mucosal lesions. GSDMD‐N is a key cell membrane pore‐forming protein that facilitates the release of inflammatory mediators, and the activation of cleaved GSDMD‐N and inflammatory factors is triggered by cleaved caspase‐1.^61^, ^62^ In response to HIF‐1α‐mediated ROS release from dysfunctional mitochondria under hypoxia, the NLRP3 inflammasome is swiftly activated, and IL‐18 and IL‐1β are secreted with the assistance of cleaved caspase‐1 and GSDMD‐N, which boosts extracellular inflammation and eventually aggravates gastric mucosal inflammatory lesions in PHT and promotes the proliferation of gastric carcinoma cells. Whether and how other elements or events, such as PKM2, ATP and K^+^ efflux, and disturbed mitochondrial Ca^2+^ homeostasis activate the NLRP3 inflammasome under hypoxia in PHG and GC tissues need to be elucidated in our future work.

In conclusion, we revealed that HIF‐1α expression is upregulated in the gastric mucosa in PHT and GC, which can promote METTL3/IGF2BP3‐dependent Drp1 m6A methylation under hypoxia to enhance mitochondrial fission, dysfunction and glycolysis, and that HIF‐1α can also enhance glycolysis via LDHA. Accompanying the alterations in antioxidant enzyme activity and dysfunction of the ETC, these dysfunctional mitochondria powerfully boost mtROS production to induce NLRP3 inflammasome activation and subsequent release of IL‐18 and IL‐1β via the caspase‐1/GSDMD‐N pathway. These factors form an anoxic and inflammatory microenvironment in the gastric mucosa, and the increase in inflammation exacerbates the progression of gastric mucosal lesions. This network provides a potential therapeutic target for the development of benign and malignant gastric mucosal lesions in response to hypoxic condition (Figure 10).

## AUTHOR CONTRIBUTIONS

Yuelin Xiao and Xianzhi Liu performed the mouse experiments and signalling pathway study and analysed the data. Kaiduan Xie and Jiajie Luo collected the clinical samples and performed the clinical study. Xiaoli Huang, Yiwang Zhang and Jinni Luo contributed the essential reagents and conducted the mouse and cell studies. Siwei Tan designed the whole project, supervised the research and wrote the paper. All authors have read and approved the manuscript.

## CONFLICT OF INTEREST STATEMENT

The authors declare they have no conflicts of interest.

## ETHICS STATEMENT

This study protocol was approved by the Institute Research Ethics Committee of the Third Affiliated Hospitals of Sun Yat‐Sen University (no. RG2023‐032‐01). All animal studies and experimental protocols were approved and conducted according to the Institutional Animal Care and Use Committee of South China Agricultural University (no. 2022F122).

## Supporting information

Supporting information

Supporting information

Supporting information

Supporting information

Supporting information

Supporting information

Supporting information

Supporting information

## Data Availability

All data relevant to the study are included in the article or uploaded as the Supporting Information. Other data are available upon request from the corresponding author.

## References

[1] Park MH , Choi KY , Jung Y , Min DS . Phospholipase D1 protein coordinates dynamic assembly of HIF‐1α‐PHD‐VHL to regulate HIF‐1α stability. Oncotarget. 2014;5:11857‐11872. doi:10.18632/oncotarget.2613 25361009 PMC4323006

[2] Wang XH , Jiang ZH , Yang HM , Zhang Y , Xu LH . Hypoxia‐induced FOXO4/LDHA axis modulates gastric cancer cell glycolysis and progression. Clin Transl Med. 2021;11:e279. doi:10.1002/ctm2.279 33463054 PMC7809603

[3] Thuluvath PJ , Yoo HY . Portal hypertensive gastropathy. Am J Gastroenterol. 2002;97:2973‐2978. doi:10.1111/j.1572-0241.2002.07094.x 12492178

[4] Lo GH . Mechanism of portal hypertensive gastropathy: an unresolved issue. J Gastroenterol Hepatol. 2009;24:1482‐1483. doi:10.1111/j.1440-1746.2009.06012.x 19743994

[5] Portal hypertensive gastropathy. Lancet. 1991;338:1045‐1046. 1681359

[6] Hu CJ , Wang LY , Chodosh LA , Keith B , Simon MC . Differential roles of hypoxia‐inducible factor 1alpha (HIF‐1alpha) and HIF‐2alpha in hypoxic gene regulation. Mol Cell Biol. 2003;23:9361‐9374. doi:10.1128/mcb.23.24.9361-9374.2003 14645546 PMC309606

[7] Aberg KA , McClay JL , Nerella S , et al. Methylome‐wide association study of schizophrenia: identifying blood biomarker signatures of environmental insults. JAMA Psychiatry. 2014;71:255‐264. doi:10.1001/jamapsychiatry.2013.3730 24402055 PMC4331014

[8] Wang K , Liu R , Li J , et al. Quercetin induces protective autophagy in gastric cancer cells: involvement of Akt‐mTOR‐ and hypoxia‐induced factor 1α‐mediated signaling. Autophagy. 2011;7:966‐978. doi:10.4161/auto.7.9.15863 21610320

[9] Jain IH , Zazzeron L , Goli R , et al. Hypoxia as a therapy for mitochondrial disease. Science. 2016;352:54‐61. doi:10.1126/science.aad9642 26917594 PMC4860742

[10] Song IS , Wang AG , Yoon SY , et al. Regulation of glucose metabolism‐related genes and VEGF by HIF‐1alpha and HIF‐1beta, but not HIF‐2alpha, in gastric cancer. Exp Mol Med. 2009;41:51‐58. doi:10.3858/emm.2009.41.1.007 19287200 PMC2679279

[11] Gong W , Xu J , Wang Y , et al. Nuclear genome‐derived circular RNA circPUM1 localizes in mitochondria and regulates oxidative phosphorylation in esophageal squamous cell carcinoma. Signal Transduct Target Ther. 2022;7:40. doi:10.1038/s41392-021-00865-0 35153295 PMC8841503

[12] Greuter T , Yaqoob U , Gan C , et al. Mechanotransduction‐induced glycolysis epigenetically regulates a CXCL1‐dominant angiocrine signaling program in liver sinusoidal endothelial cells in vitro and in vivo. J Hepatol. 2022;77:723‐734. doi:10.1016/j.jhep.2022.03.029 35421427 PMC9391258

[13] Chang J , Wu H , Wu J , et al. Constructing a novel mitochondrial‐related gene signature for evaluating the tumor immune microenvironment and predicting survival in stomach adenocarcinoma. J Transl Med. 2023;21:191. doi:10.1186/s12967-023-04033-6 36915111 PMC10012538

[14] Wallace DC , Fan W , Procaccio V . Mitochondrial energetics and therapeutics. Annu Rev Pathol. 2010;5:297‐348. doi:10.1146/annurev.pathol.4.110807.092314 20078222 PMC3245719

[15] Guo L , Cui C , Wang J , et al. PINCH‐1 regulates mitochondrial dynamics to promote proline synthesis and tumor growth. Nat Commun. 2020;11:4913. doi:10.1038/s41467-020-18753-6 33004813 PMC7529891

[16] Yan T , Zhao Y . Acetaldehyde induces phosphorylation of dynamin‐related protein 1 and mitochondrial dysfunction via elevating intracellular ROS and Ca^2+^ levels. Redox Biol. 2020;28:101381. doi:10.1016/j.redox.2019.101381 31756635 PMC6879985

[17] Li T , Han J , Jia L , Hu X , Chen L , Wang Y . PKM2 coordinates glycolysis with mitochondrial fusion and oxidative phosphorylation. Protein Cell. 2019;10:583‐594. doi:10.1007/s13238-019-0618-z 30887444 PMC6626593

[18] Giedt RJ , Pfeiffer DR , Matzavinos A , Kao CY , Alevriadou BR . Mitochondrial dynamics and motility inside living vascular endothelial cells: role of bioenergetics. Ann Biomed Eng. 2012;40:1903‐1916. doi:10.1007/s10439-012-0568-6 22527011 PMC3416955

[19] Prieto J , León M , Ponsoda X , et al. Early ERK1/2 activation promotes DRP1‐dependent mitochondrial fission necessary for cell reprogramming. Nat Commun. 2016;7:11124. doi:10.1038/ncomms11124 27030341 PMC4821885

[20] Barsoum MJ , Yuan H , Gerencser AA , et al. Nitric oxide‐induced mitochondrial fission is regulated by dynamin‐related GTPases in neurons. EMBO J. 2006;25:3900‐3911. doi:10.1038/sj.emboj.7601253 16874299 PMC1553198

[21] Tanoue K , Hashizume M , Wada H , Ohta M , Kitano S , Sugimachi K . Effects of endoscopic injection sclerotherapy on portal hypertensive gastropathy: a prospective study. Gastrointest Endosc. 1992;38:582‐585. doi:10.1016/s0016-5107(92)70522-7 1397916

[22] Tan S , Li L , Chen T , et al. β‐Arrestin‐1 protects against endoplasmic reticulum stress/p53‐upregulated modulator of apoptosis‐mediated apoptosis via repressing p‐p65/inducible nitric oxide synthase in portal hypertensive gastropathy. Free Radical Biol Med. 2015;87:69‐83. doi:10.1016/j.freeradbiomed.2015.06.004 26119788

[23] Tan S , Xu M , Ke B , et al. IL‐6‐driven FasL promotes NF‐κBp65/PUMA‐mediated apoptosis in portal hypertensive gastropathy. Cell Death Dis. 2019;10:748. doi:10.1038/s41419-019-1954-x 31582729 PMC6776649

[24] Tan S , Chen X , Xu M , et al. PGE(2)/EP(4) receptor attenuated mucosal injury via β‐arrestin1/Src/EGFR‐mediated proliferation in portal hypertensive gastropathy. Br J Pharmacol. 2017;174:848‐866. doi:10.1111/bph.13752 28213942 PMC5386997

[25] Liu X , Tan S , Liu H , et al. Hepatocyte‐derived MASP1‐enriched small extracellular vesicles activate HSCs to promote liver fibrosis. Hepatology. 2023;77:1181‐1197. doi:10.1002/hep.32662 35849032

[26] Ji WK , Chakrabarti R , Fan X , Schoenfeld L , Strack S , Higgs HN . Receptor‐mediated Drp1 oligomerization on endoplasmic reticulum. J Cell Biol. 2017;216:4123‐4139. doi:10.1083/jcb.201610057 29158231 PMC5716263

[27] Wong YC , Kim S , Cisneros J , et al. Mid51/Fis1 mitochondrial oligomerization complex drives lysosomal untethering and network dynamics. J Cell Biol. 2022;221:e202206140. doi:10.1083/jcb.202206140 36044022 PMC9437119

[28] Yu R , Jin SB , Ankarcrona M , Lendahl U , Nistér M , Zhao J . The molecular assembly state of Drp1 controls its association with the mitochondrial recruitment receptors Mff and MIEF1/2. Front Cell Dev Biol. 2021;9:706687. doi:10.3389/fcell.2021.706687 34805137 PMC8602864

[29] Noto JM , Piazuelo MB , Romero‐Gallo J , et al. Targeting hypoxia‐inducible factor‐1 alpha suppresses *Helicobacter pylori*‐induced gastric injury via attenuation of both cag‐mediated microbial virulence and proinflammatory host responses. Gut Microbes. 2023;15:2263936. doi:10.1080/19490976.2023.2263936 37828903 PMC10578190

[30] He C , Wang L , Zhang J , Xu H . Hypoxia‐inducible microRNA‐224 promotes the cell growth, migration and invasion by directly targeting RASSF8 in gastric cancer. Mol Cancer. 2017;16:35. doi:10.1186/s12943-017-0603-1 28173803 PMC5297251

[31] Gana JC , Cifuentes LI , Gattini D , et al. Band ligation versus beta‐blockers for primary prophylaxis of oesophageal variceal bleeding in children with chronic liver disease or portal vein thrombosis. Cochrane Database Syst Rev. 2019;9:Cd010546. doi:10.1002/14651858.CD010546.pub2 31550050 PMC6758973

[32] Zhang Y , Lu H , Ji H , Li Y . p53 upregulated by HIF‐1α promotes gastric mucosal epithelial cells apoptosis in portal hypertensive gastropathy. Digestive Liver Disease. 2023;55:81‐92. doi:10.1016/j.dld.2022.06.016 35780066

[33] Yang Q , Lei X , He J , et al. N4‐acetylcytidine drives glycolysis addiction in gastric cancer via NAT10/SEPT9/HIF‐1α positive feedback loop. Adv Sci. 2023;10:e2300898. doi:10.1002/advs.202300898 PMC10427357 37328448

[34] Li S , Li W , Yuan J , et al. Impaired oxygen‐sensitive regulation of mitochondrial biogenesis within the von Hippel‒Lindau syndrome. Nat Metab. 2022;4:739‐758. doi:10.1038/s42255-022-00593-x 35760869 PMC9236906

[35] Xie L , Shi F , Li Y , et al. Drp1‐dependent remodeling of mitochondrial morphology triggered by EBV‐LMP1 increases cisplatin resistance. Signal Transduct Target Ther. 2020;5:56. doi:10.1038/s41392-020-0151-9 32433544 PMC7237430

[36] Kornfeld OS , Qvit N , Haileselassie B , Shamloo M , Bernardi P , Mochly‐Rosen D . Interaction of mitochondrial fission factor with dynamin related protein 1 governs physiological mitochondrial function in vivo. Sci Rep. 2018;8:14034. doi:10.1038/s41598-018-32228-1 30232469 PMC6145916

[37] Hu L , Ding M , Tang D , et al. Targeting mitochondrial dynamics by regulating Mfn2 for therapeutic intervention in diabetic cardiomyopathy. Theranostics. 2019;9:3687‐3706. doi:10.7150/thno.33684 31281507 PMC6587356

[38] Yue SW , Liu HL , Su HF , et al. m6A‐regulated tumor glycolysis: new advances in epigenetics and metabolism. Mol Cancer. 2023;22:137. doi:10.1186/s12943-023-01841-8 37582735 PMC10426175

[39] Xu W , Lai Y , Pan Y , et al. m6A RNA methylation‐mediated NDUFA4 promotes cell proliferation and metabolism in gastric cancer. Cell Death Dis. 2022;13:715. doi:10.1038/s41419-022-05132-w 35977935 PMC9385701

[40] Tu B , Song K , Zhou Y , et al. METTL3 boosts mitochondrial fission and induces cardiac fibrosis by enhancing LncRNA GAS5 methylation. Pharmacol Res. 2023;194:106840. doi:10.1016/j.phrs.2023.106840 37379961

[41] Zhang Y , Liu Y , Xie Z , et al. Inhibition of PFKFB preserves intestinal barrier function in sepsis by inhibiting NLRP3/GSDMD. Oxid Med Cell Long. 2022;2022:8704016. doi:10.1155/2022/8704016 PMC9803577 36589684

[42] Liu X , Wang X , Zhang J , et al. Warburg effect revisited: an epigenetic link between glycolysis and gastric carcinogenesis. Oncogene. 2010;29:442‐450. doi:10.1038/onc.2009.332 19881551

[43] Li C , Lu C , Gong L , et al. SHP2/SPI1 axis promotes glycolysis and the inflammatory response of macrophages in *Helicobacter pylori*‐induced pediatric gastritis. Helicobacter. 2022;27:12895. doi:10.1111/hel.12895 35437862

[44] Cheng CT , Kuo CY , Ouyang C , et al. Metabolic stress‐induced phosphorylation of KAP1 Ser473 blocks mitochondrial fusion in breast cancer cells. Cancer Res. 2016;76:5006‐5018. doi:10.1158/0008-5472.Can-15-2921 27364555 PMC5316485

[45] Lu X , Kang Y . Hypoxia and hypoxia‐inducible factors: master regulators of metastasis. Clin Cancer Res. 2010;16:5928‐5935. doi:10.1158/1078-0432.Ccr-10-1360 20962028 PMC3005023

[46] Xu R , Yuan LS , Gan YQ , et al. Potassium ion efflux induces exaggerated mitochondrial damage and non‐pyroptotic necrosis when energy metabolism is blocked. Free Radical Biol Med. 2024;212:117‐132. doi:10.1016/j.freeradbiomed.2023.12.029 38151213

[47] Nolfi‐Donegan D , Braganza A , Shiva S . Mitochondrial electron transport chain: oxidative phosphorylation, oxidant production, and methods of measurement. Redox Biol. 2020;37:101674. doi:10.1016/j.redox.2020.101674 32811789 PMC7767752

[48] Kahveci AS , Barnatan TT , Kahveci A , et al. Oxidative stress and mitochondrial abnormalities contribute to decreased endothelial nitric oxide synthase expression and renal disease progression in early experimental polycystic kidney disease. Int J Mol Sci. 2020;21:1994. doi:10.3390/ijms21061994 32183375 PMC7139316

[49] Rohwer N , Cramer T . Hypoxia‐mediated drug resistance: novel insights on the functional interaction of HIFs and cell death pathways. Drug Resist Updat. 2011;14:191‐201. doi:10.1016/j.drup.2011.03.001 21466972

[50] Hong Z , Wang H , Zhang T , et al. The HIF‐1/BNIP3 pathway mediates mitophagy to inhibit the pyroptosis of fibroblast‐like synoviocytes in rheumatoid arthritis. Int Immunopharmacol. 2023;127:111378. doi:10.1016/j.intimp.2023.111378 38141408

[51] Zhang W , Xia CL , Qu YD , et al. MicroRNA‐18a regulates the Pyroptosis, Apoptosis, and Necroptosis (PANoptosis) of osteoblasts induced by tumor necrosis factor‐α via hypoxia‐inducible factor‐1α. Int Immunopharmacol. 2024;128:111453. doi:10.1016/j.intimp.2023.111453 38241841

[52] Sha JF , Xie QM , Chen N , et al. TLR2‐hif1α‐mediated glycolysis contributes to pyroptosis and oxidative stress in allergic airway inflammation. Free Radical Biol Med. 2023;200:102‐116. doi:10.1016/j.freeradbiomed.2023.03.007 36907255

[53] Karki R , Man SM , Kanneganti TD . Inflammasomes and Cancer. Cancer Immunol Res. 2017;5:94‐99. doi:10.1158/2326-6066.Cir-16-0269 28093447 PMC5593081

[54] Zhou R , Yazdi AS , Menu P , Tschopp J . A role for mitochondria in NLRP3 inflammasome activation. Nature. 2011;469:221‐225. doi:10.1038/nature09663 21124315

[55] Grootjans J , Kaser A , Kaufman RJ , Blumberg RS . The unfolded protein response in immunity and inflammation. Nat Rev Immunol. 2016;16:469‐484. doi:10.1038/nri.2016.62 27346803 PMC5310224

[56] Zhong Z , Liang S , Sanchez‐Lopez E , et al. New mitochondrial DNA synthesis enables NLRP3 inflammasome activation. Nature. 2018;560:198‐203. doi:10.1038/s41586-018-0372-z 30046112 PMC6329306

[57] Zeng B , Huang Y , Chen S , et al. Dextran sodium sulfate potentiates NLRP3 inflammasome activation by modulating the KCa3.1 potassium channel in a mouse model of colitis. Cell Mol Immunol. 2022;19:925‐943. doi:10.1038/s41423-022-00891-0 35799057 PMC9338299

[58] Shimada K , Crother TR , Karlin J , et al. Oxidized mitochondrial DNA activates the NLRP3 inflammasome during apoptosis. Immunity. 2012;36:401‐414. doi:10.1016/j.immuni.2012.01.009 22342844 PMC3312986

[59] Xie M , Yu Y , Kang R , et al. PKM2‐dependent glycolysis promotes NLRP3 and AIM2 inflammasome activation. Nat Commun. 2016;7:13280. doi:10.1038/ncomms13280 27779186 PMC5093342

[60] Palsson‐McDermott EM , Curtis AM , Goel G , et al. Pyruvate kinase M2 regulates Hif‐1α activity and IL‐1β induction and is a critical determinant of the warburg effect in LPS‐activated macrophages. Cell Metab. 2015;21:65‐80. doi:10.1016/j.cmet.2014.12.005 25565206 PMC5198835

[61] Gram AM , Booty LM , Bryant CE . Chopping GSDMD: caspase‐8 has joined the team of pyroptosis‐mediating caspases. EMBO J. 2019;38:e102065. doi:10.15252/embj.2019102065 30988015 PMC6517822

[62] Voet S , Srinivasan S , Lamkanfi M , van Loo G . Inflammasomes in neuroinflammatory and neurodegenerative diseases. EMBO Mol Med. 2019;11:e10248. doi:10.15252/emmm.201810248 31015277 PMC6554670

