# Supplementary figures and images for: Mitochondrial dysfunction induced by HIF‐1α under hypoxia contributes to the development of gastric mucosal lesions
Source: Clin Transl Med. 2024 Apr 15;14(4):e1653. doi: 10.1002/ctm2.1653 (PMC11016940; doi:10.1002/ctm2.1653)

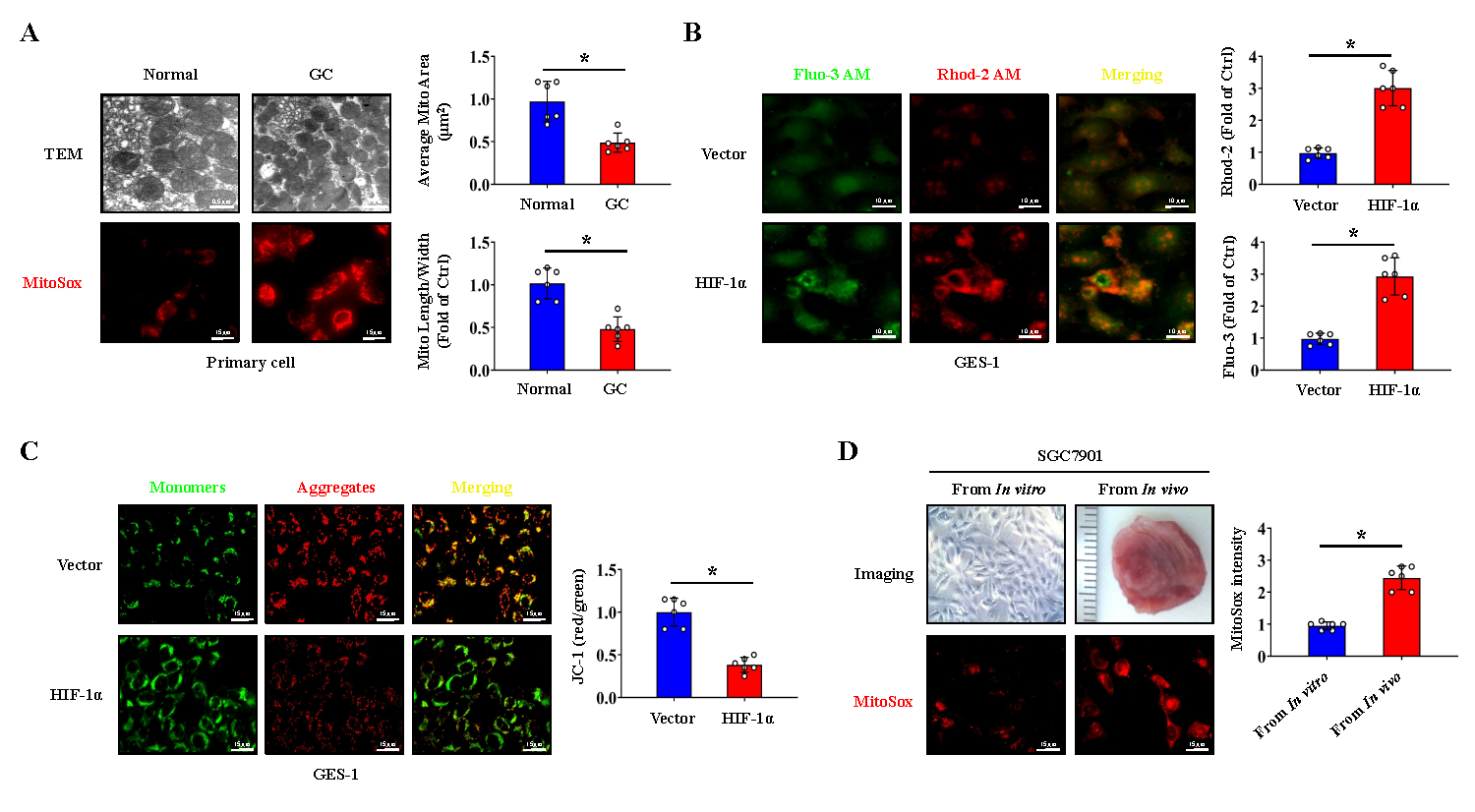

Supplement: Supplementary file 1 — Supporting information [file CTM2-14-e1653-s002.tif]

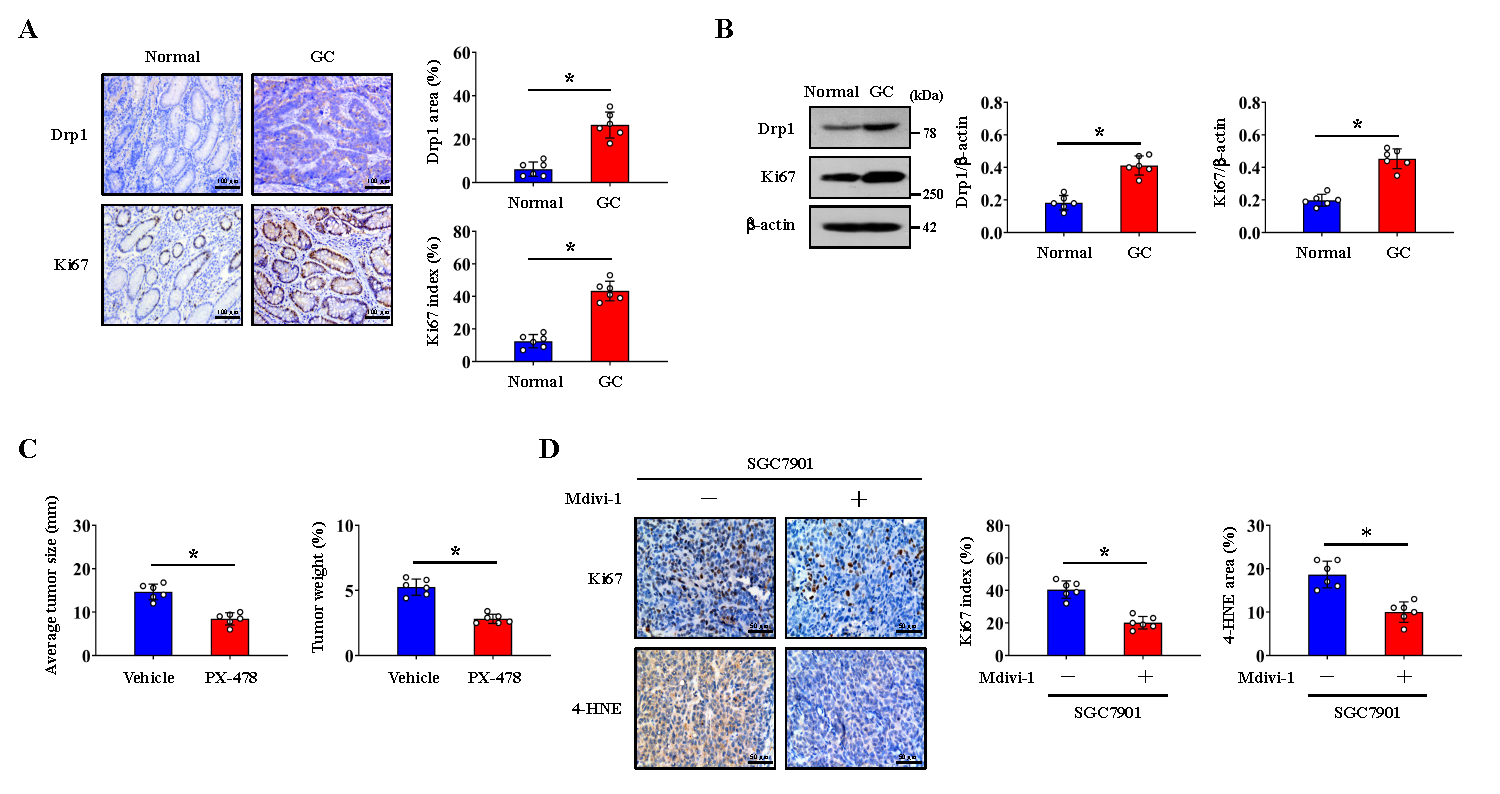

Supplement: Supplementary file 2 — Supporting information [file CTM2-14-e1653-s005.tif]

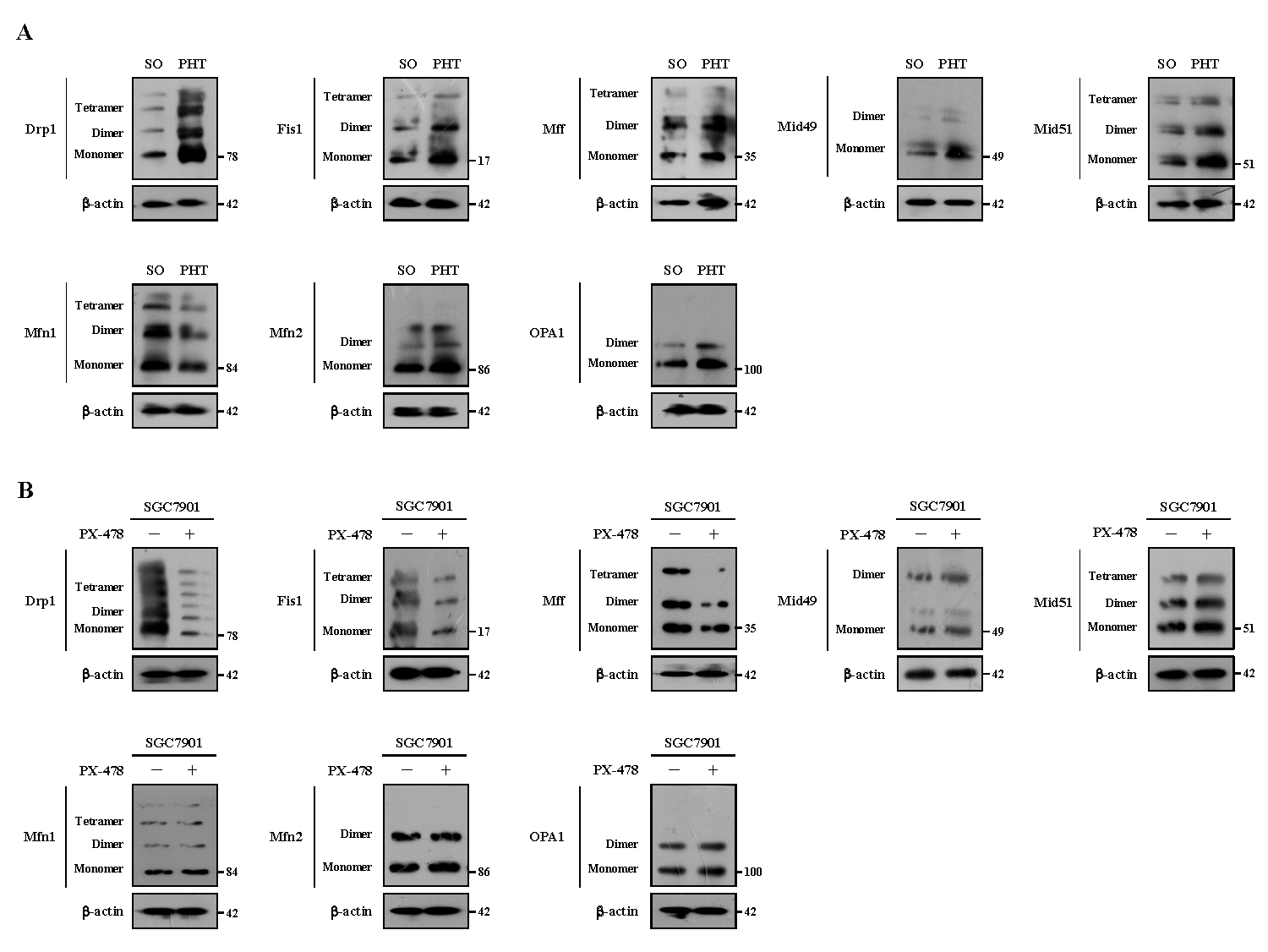

Supplement: Supplementary file 3 — Supporting information [file CTM2-14-e1653-s008.tif]

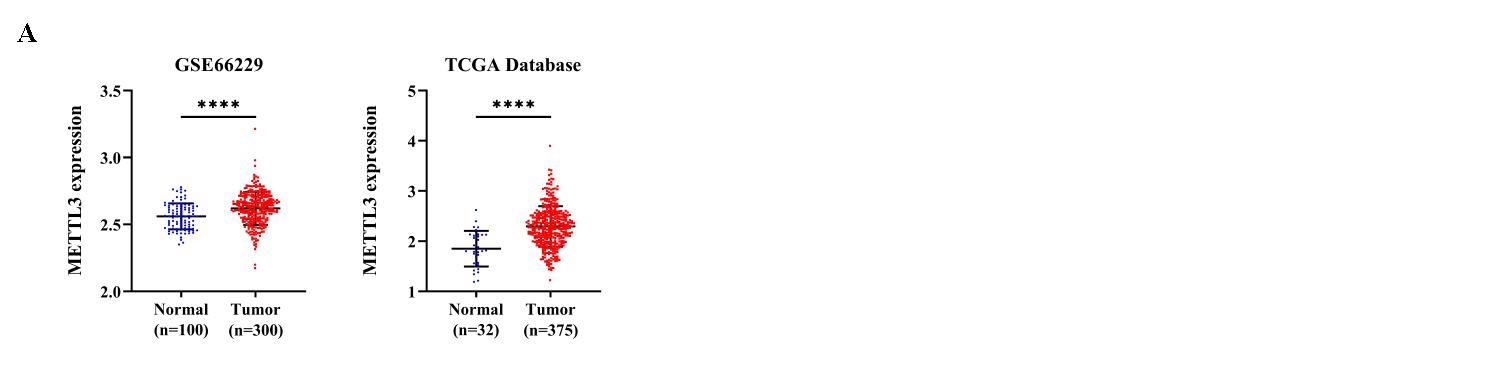

Supplement: Supplementary file 4 — Supporting information [file CTM2-14-e1653-s004.tif]

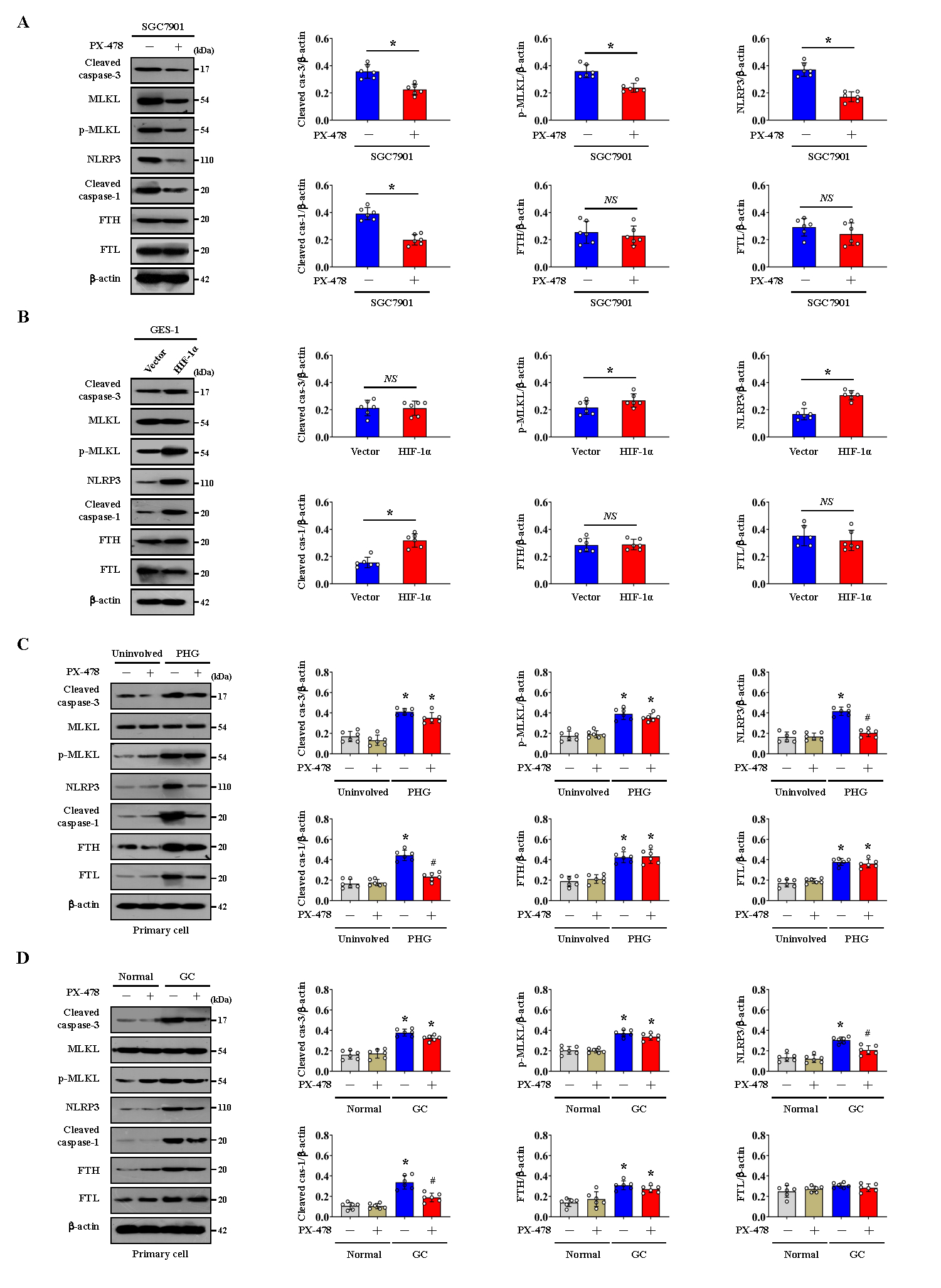

Supplement: Supplementary file 5 — Supporting information [file CTM2-14-e1653-s006.tif]

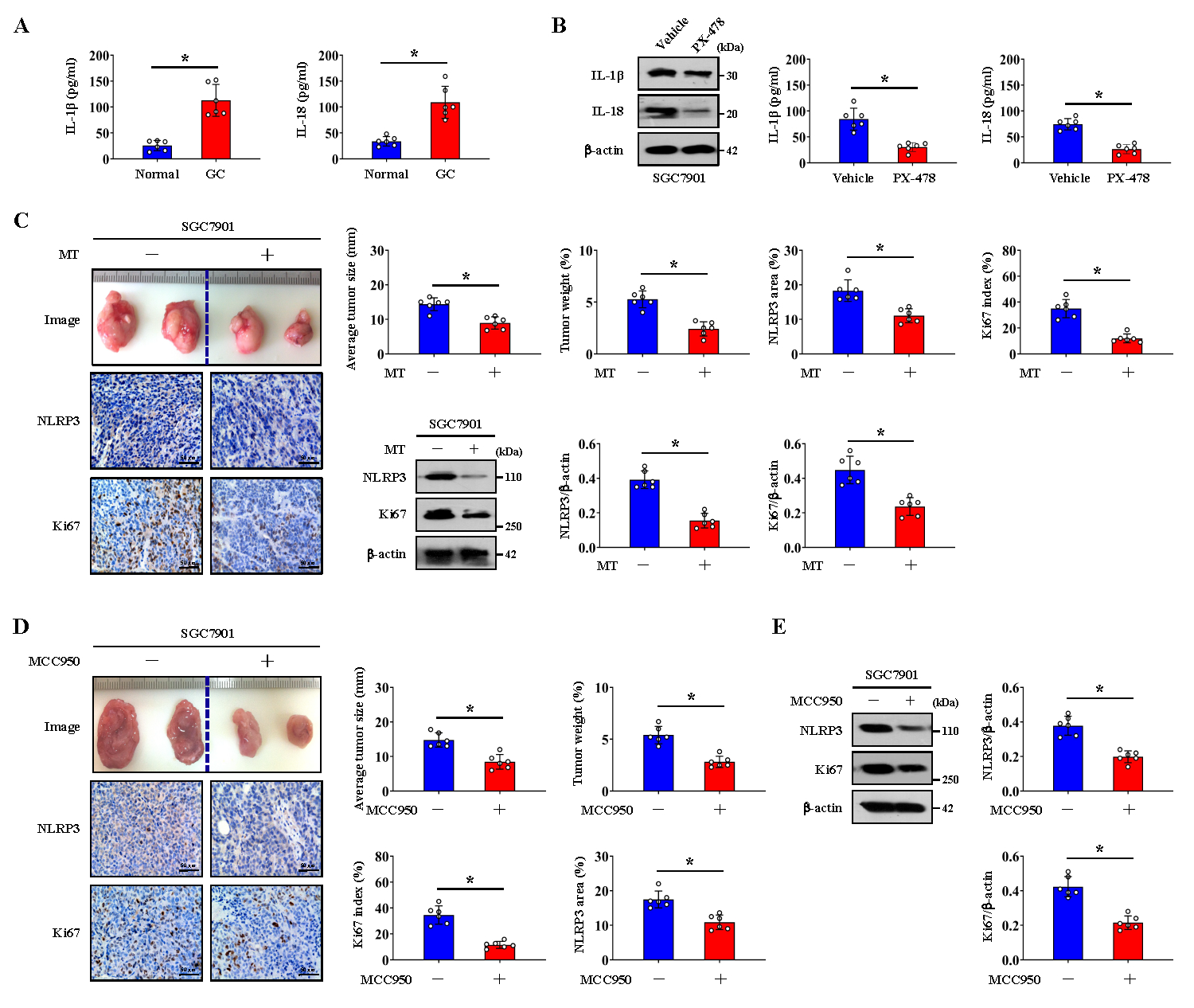

Supplement: Supplementary file 6 — Supporting information [file CTM2-14-e1653-s001.tif]
