# Supplementary material for: Mitochondrial dysfunction induced by HIF‐1α under hypoxia contributes to the development of gastric mucosal lesions
Source: Clin Transl Med. 2024 Apr 15;14(4):e1653. doi: 10.1002/ctm2.1653 (PMC11016940; doi:10.1002/ctm2.1653)
Supplement: Supplementary file 7 — Supporting information [file CTM2-14-e1653-s007.docx]

**Supplemental Figure 1. HIF-1α participates in mitochondrial dysfunction under hypoxia in both PHG and GC.**

1. TEM image and MitoSox staining (isolated primary cells) of the normal and GC groups (n = 6 per group). The quantified mitochondrial area and the ratio of mitochondrial length to width were analyzed via TEM. **P* < 0.05. (B) The cytosolic and mitochondrial Ca^2+^ levels in GES-1 cells transfected with vector or *HIF-1α* were measured with Fluo-3 AM (green) and Rhod-2 (red), respectively. Merged images (yellow) were also shown (n = 6 per group, **P* < 0.05). (C) JC-1 staining of vector- and *HIF-1α*-transfected GES-1 cells. The histogram showed the ratio of JC-1 (n = 6 per group, **P* < 0.05). (D) Representative photographs of SGC7901 cell morphology *in vitro* and isolated xenograft tumors *in vivo*. The MitoSox intensity of the indicated cells was also determined (n = 6 per group, **P* < 0.05).

Abbreviations: PHG, portal hypertensive gastropathy; SO, sham operation; PHT, portal hypertension; GC, gastric cancer; TEM, transmission electron microscopy; NS, not significant.

**Supplemental Figure 2. Drp1-dependent mitochondrial fission is involved in the development of GC.**

1. Drp1 and Ki67 IHC staining (brown) and area percentage analyses of normal gastric and GC tissues. n = 6 per group, **P* < 0.05. (B) The expression of Drp1 and Ki67 in the normal and GC groups was measured by western blotting. The densitometry units of Drp1/β-actin and Ki67/β-actin were also shown. n = 6 per group, **P* < 0.05. (C) The average tumor size and tumor weight from Figure 2F. n = 6 per group, **P* < 0.05. (D) Ki67 and 4-HNE IHC staining (brown) of the gastric cancer tissues of SGC7901 xenografts from nude mice with Mdivi-1 treatment or not. The Ki67 index (%) and 4-HNE area (%) were examined. n = 6 per group, **P* < 0.05.

Abbreviations: GC, gastric cancer.

**Supplemental Figure 3. Enhanced Drp1 and Fis1 oligomerization contributes to mitochondrial fission in both PHT and GC.**

(A) The oligomeric states of Drp1, Fis1, Mff, Mid49, Mid51, Mfn1, Mfn2 and OPA1 in the SO and PHT mouse groups were analyzed by western blotting with the appropriate antibodies. (B) The oligomeric states of Drp1, Fis1, Mff, Mid49, Mid51, Mfn1, Mfn2 and OPA1 in cells isolated from SGC7901 xenografts in nude mice (with or without PX-478 treatment) were analyzed by western blotting with the indicated antibodies.

Abbreviations: SO, sham operation; PHT, portal hypertension; GC, gastric cancer; Drp1, dynamin-related protein 1; Fis1, fission protein 1; Mff, mitochondrial fission factor; Mid49, mitochondrial dynamics protein 49; Mid51, mitochondrial dynamics protein 51; Mfn1, mitofusin 1; Mfn2, mitofusin 2; OPA1, optic atrophy 1.

**Supplemental Figure 4. METTL3 is overexpressed in GC patients.**

(A) The expression of the m6A-modifying enzyme METTL3 in GC tissues (n = 300) compared with that in normal gastric tissues (n = 100) was analyzed with the GEO dataset GSE66229 after excluding samples without clinical information (https://www.ncbi.nlm.nih.gov/geo/). RNA-seq data from 375 gastric tumor samples and 32 normal gastric tissue samples were also obtained from the published clinical dataset TCGA to validate the difference in METTL3 expression between tumor and normal tissues (https://portal.gdc.cancer.gov).

Abbreviations: TCGA, The Cancer Genome Atlas; GEO, Gene Expression Omnibus; GC, gastric cancer.

**Supplemental Figure 5. NLRP3 inflammasome-mediated pyroptosis mainly contributes to the development of gastric mucosal lesions modulated by HIF-1α signaling.**

(A) The protein levels of cleaved caspase-3, MLKL, p-MLKL, NLRP3, cleaved caspase-1, FTH and FTL in cells isolated from SGC7901 xenografts in nude mice (with or without PX-478 treatment) were analyzed by western blotting. The ratios of densitometric units of the normalized cleaved caspase-3/β-actin, p-MLKL/β-actin, NLRP3/β-actin, cleaved caspase-1/β-actin, FTH/β-actin and FTL/β-actin were also analyzed. n = 6 per group. **P* < 0.05. *NS*, not significant. (B) The cleaved caspase-3, MLKL, p-MLKL, NLRP3, cleaved caspase-1, FTH and FTL levels in vector- and *HIF-1α*-transfected GES-1 cells were determined by western blotting. The ratios of densitometric units of the normalized cleaved caspase-3/β-actin, p-MLKL/β-actin, NLRP3/β-actin, cleaved caspase-1/β-actin, FTH/β-actin and FTL/β-actin were further analyzed. n = 6 per group. **P* < 0.05. *NS*, not significant. (C) Primary epithelial cells isolated from PHG patients and healthy volunteers (uninvolved) were cultured in RPMI 1640 medium and then treated with or without PX-478. The cleaved caspase-3, MLKL, p-MLKL, NLRP3, cleaved caspase-1, FTH and FTL levels were measured by western blotting. The ratios of densitometric units of the normalized cleaved caspase-3/β-actin, p-MLKL/β-actin, NLRP3/β-actin, cleaved caspase-1/β-actin, FTH/β-actin and FTL/β-actin were further analyzed. n = 6 per group. **P* < 0.05 versus cells from the uninvolved group, #*P* < 0.05 versus cells not treated with PX-478 in the PHG group. (D) Primary cells isolated from normal and GC tissues were cultured in RPMI 1640 medium and then treated with or without PX-478. The cleaved caspase-3, MLKL, p-MLKL, NLRP3, cleaved caspase-1, FTH and FTL levels in the above cells were measured by western blotting. The ratios of densitometric units of the normalized cleaved caspase-3/β-actin, p-MLKL/β-actin, NLRP3/β-actin, cleaved caspase-1/β-actin, FTH/β-actin and FTL/β-actin were further analyzed. n = 6 per group. **P* < 0.05 versus cells from the normal group, #*P* < 0.05 versus cells not treated with PX-478 in the GC group.

Abbreviations: PHG, portal hypertensive gastropathy; SO, sham operation; PHT, portal hypertension; GC, gastric cancer; NS, not significant.

**Supplemental Figure 6. Mitochondrial dysfunction-induced oxidative stress activates the NLRP3 inflammasome to contribute to the development of GC.**

1. The levels of IL-1β and IL-18 in the normal and GC groups were shown. n = 6 per group, **P* < 0.05. (B) Protein analyses of IL-1β and IL-18 in the SGC7901 xenografts of nude with or without PX-478 treatment. n = 6 per group, **P* < 0.05. (C) Representative images of general gastric tumor tissue and IHC staining for NLRP3 (brown) and Ki67 (brown) in SGC7901 mouse gastric tumor model mice with or without MT treatment. The average tumor size, tumor weight, NLRP3 area (%) and Ki67 index (%) were shown. The protein expression of NLRP3 and Ki67 in the above groups was also measured by western blotting. n = 6 per group, **P* < 0.05. (D) Representative images of the general organization of gastric tumor tissue and IHC staining for NLRP3 (brown) and Ki67 (brown) in SGC7901 mice with or without MCC950 treatment. The average tumor size, tumor weight, relative number of NLRP3-positive areas (%) and Ki67 index (%) were also shown. n = 6 per group, **P* < 0.05. (E) The levels of NLRP3 and Ki67 in the indicated groups were determined by western blotting. The ratios of densitometry units of normalized NLRP3 and Ki67 to β-actin were determined. n = 6 per group. **P* < 0.05.

Abbreviations: GC, gastric cancer; MT, mito-TEMPO.

Yuelin Xiao^1, #^, Xianzhi Liu^1, #^, Kaiduan Xie^1^, Jiajie Luo^1^, Yiwang Zhang^2^, Xiaoli Huang^1^, Jinni Luo^1^, Siwei Tan^1,^ *

^1^Department of Gastroenterology, the Third Affiliated Hospital of Sun Yat-sen University, Guangzhou, Guangdong Province, 510630, China

^2^Department of Pathology, the Third Affiliated Hospital of Sun Yat-sen University, Guangzhou, Guangdong Province, 510630, China

^#^Yuelin Xiao and Xianzhi Liu contributed equally to this work.

*Corresponding author: Siwei Tan, Department of Gastroenterology, the Third Affiliated Hospital of Sun Yat-sen University, 600 Tianhe Road, Guangzhou 510630, China. Tel: +86-20-85253095; Email: tansw@mail.sysu.edu.cn; xiaodatou520@sina.com.

**Acknowledgments**

We thank the Institutional Animal Care and Use Committee of South China Agricultural University for providing the mouse breeding and experimental studies in this study. We also thank the Institutional Animal Care and Use Committee of the Third Affiliated Hospital of Sun Yat-sen University for providing mouse experimental studies and technical support for this experiment. This work was supported by grants from the National Natural Science Foundation of China (82170569); the Natural Science Foundation of Guangdong Province (2022A1515012546, 2023A1515011204); the Science and Technology Planning Projects of Guangzhou City (2024A04J6565); and the Major Talent Project Training Program of the Third Affiliated Hospital of Sun Yat-sen University (P02089).

**Conflicts of interest**

The authors declare that they have no conflicts of interest.

**Author contributions**

Yuelin Xiao and Xianzhi Liu performed the mouse experiments and signaling pathway study and analyzed the data; Kaiduan Xie and Jiajie Luo collected the clinical samples and performed the clinical study; Xiaoli Huang, Yiwang Zhang and Jinni Luo contributed the essential reagents and conducted the mouse and cell studies; and Siwei Tan designed the whole project, supervised the research and wrote the paper. All authors have read and approved the manuscript.
